# New online in-air signature recognition dataset and embodied cognition inspired feature selection

**DOI:** 10.1038/s41598-025-03917-5

**Published:** 2025-06-02

**Authors:** Yuheng Guo, Yuhan Zhou, Yifan Ge, Junwei Yu, Gen Li, Hiroyuki Sato

**Affiliations:** 1 Electrical Engineering and Information Systems, The University of Tokyo, 7-chōme-3-1 Hongō, Bunkyo, 113-8654 Tokyo Japan; 2 Information Systems Architecture Science, National Institute of Informatics, 2 Chome-1-2 Hitotsubashi, Chiyoda, 101-0003 Tokyo Japan; 3 School of Mechanical and Manufacturing Engineering, University of New South Wales, High St, Sydney, NSW 2052 Australia; 4 Department of Gerontology, University of Southampton, Burgess Rd, Southampton, Hampshire SO17 1BJ UK; 5 Kyushu University, 744 Motooka Nishi-ku, Nishi Ward, Fukuoka, 819-0395 Japan

**Keywords:** Online signature, Dataset, Feature selection, Deep learning, Inertial data, Engineering, Mathematics and computing

## Abstract

In this study, we introduce MIAS-427, one of the largest and most comprehensive inertial datasets for in-air signature recognition, comprising 4270 multivariate signals. This dataset addresses a critical gap in the field by providing a robust foundation for advancing research in cognitive computation and biometric authentication. Leveraging embodied cognition theory, we propose a novel feature selection approach using dimension-wise Shapley Value analysis, which uncovers the intrinsic relationship between human motoric preferences and device-specific sensor data. Our methodology includes a thorough statistical analysis with domain descriptors and DTW algorithms, alongside a comparative evaluation of seven deep-learning models on both the MIAS-427 and smartwatch datasets. The FCN and InceptionTime models achieved remarkable accuracies of 98% and 97.73% on MIAS-427 and smartwatch data, respectively. Notably, our analysis revealed that $gyr_y$ and $acc_x$ contributed the most (12.82%) and least (8.71%) for the smartwatch, while $att_y$ and $att_x$ contributed the most (15.63%) and least (7.26%) for MIAS-427, highlighting significant dimension compatibility variations across devices. This research not only provides a valuable dataset for the community but also offers novel insights into human motoric behavior, paving the way for the development of more effective cognitive computation models.

## Introduction

**Fig. 1:**
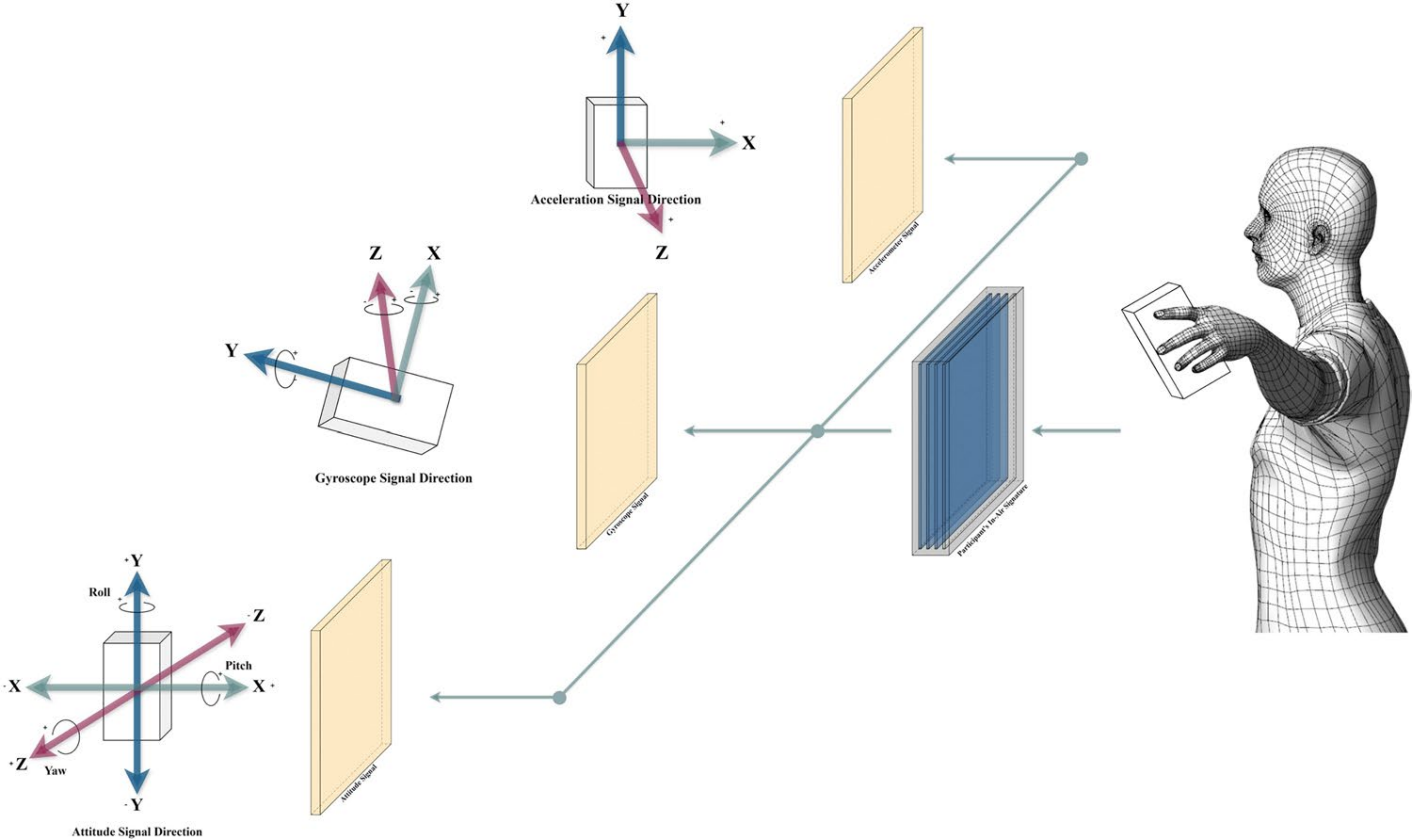
In-air signature illustration. Participant hold the phone in the air while the 3 embedded sensors record signals in 9 dimensions.

Signature recognition has long been a cornerstone of biometric authentication, with on-surface signature recognition being the most widely studied and deployed approach. On-surface signatures are captured using devices like digitized pens or touchscreens, which record the spatial and temporal dynamics of a signature as it is written on a surface. This method has proven effective in controlled environments but faces limitations in remote or contactless scenarios, where physical contact with a surface is impractical. In contrast, in-air signature recognition leverages motion sensors in portable devices, like smartphones and smartwatches^1^, to capture signatures as users write their names in the air. This contactless approach offers greater flexibility and convenience, making it particularly suitable for remote authentication and modern human-computer interaction systems^2^. Figure 1 illustrates the process of in-air signature recognition based on sensor data.

Despite its advantages, in-air signature recognition remains an understudied area, primarily due to the lack of large, high-quality datasets. Existing datasets are often limited in size and diversity, hindering the development and evaluation of robust recognition models. To address this gap, we introduce MIAS-427, a novel dataset comprising 4270 nine-dimensional in-air signature signals from 427 subjects, making it one of the largest and most comprehensive datasets in the field. MIAS-427 not only advances research in in-air signature recognition but also contributes to the broader domain of time-series classification, where the need for large, diverse datasets is critical for training and evaluating deep learning models.

Recent research has demonstrated that end-to-end deep learning models outperform traditional algorithmic methods like Dynamic Time Warping (DTW) for in-air signature recognition^3^. Building on this insight, we empirically justify the use of deep learning models for this task and evaluate seven state-of-the-art models on both the MIAS-427 dataset and smartwatch-collected in-air signatures. These models include the Fully Convolutional Network (FCN)^4,5^, Multilayer Perceptron (MLP)^4,6^, ResNet^4,7^, Encoder^8^, Multi-Channel Deep Convolutional Neural Network (MCDCNN)^9,10^, Time-CNN^11^, and InceptionTime^12^.

To further enhance our understanding of in-air signatures, we conduct a dimension-wise Shapley Value analysis to evaluate the contributions of individual sensor dimensions across multiple devices. This analysis reveals the most and least influential features, providing insights into the embodied cognition process underlying in-air signature recognition. Embodied cognition theory suggests that human motoric preferences and cognitive processes are closely tied to physical interactions with the environment. By analyzing how different sensor dimensions contribute to recognition accuracy, we demonstrate how in-air signature recognition mimics aspects of human cognition during complex motor tasks.

This study builds on our previous work on dominant and non-dominant hand signature analysis^13^ and smartwatch-based in-air signature recognition^14^. However, it introduces several key advancements:

The creation of the MIAS-427 dataset, which addresses the urgent need for large, diverse datasets in in-air signature recognition.

A systematic Shapley Value analysis to evaluate dimension-wise feature contributions across multiple devices.

A comprehensive model selection process to identify the best-performing deep learning models for large multivariate time-series data.

An exploration of the correlation between embodied cognition and in-air signature recognition, enriching the theoretical foundation for using algorithmic methods to mimic human cognitive processes.

The contributions of this work can be summarized as follows:

- We introduce MIAS-427, one of the largest datasets for in-air signature recognition and time-series classification, addressing a critical gap in the field.
- We conduct the first systematic dimension-wise Shapley Value analysis for in-air signature recognition, providing insights into feature contributions across multiple devices.
- We establish a theoretical link between embodied cognition and in-air signature recognition, shedding light on human motoric preferences and their implications for algorithmic design.

The remainder of this paper is organized as follows. “Literature review” reviews recent research in offline and online signature recognition, highlighting current challenges in the field. “Proposed online in-air signature dataset” introduces the MIAS-427 dataset and justifies the use of end-to-end deep learning models. “Time-series deep learning model structures” describes the architecture of the seven deep learning models evaluated in this study. “Embodied cognition inspired feature selection” discusses embodied cognition theory and its relevance to in-air signature recognition. “Experiment” details the experimental pipeline, while “Results” presents and discusses the results. “Research limitations and future work” outlines the limitations of this work, and “Conclusion” concludes the paper.

## Literature review

**Fig. 2:**
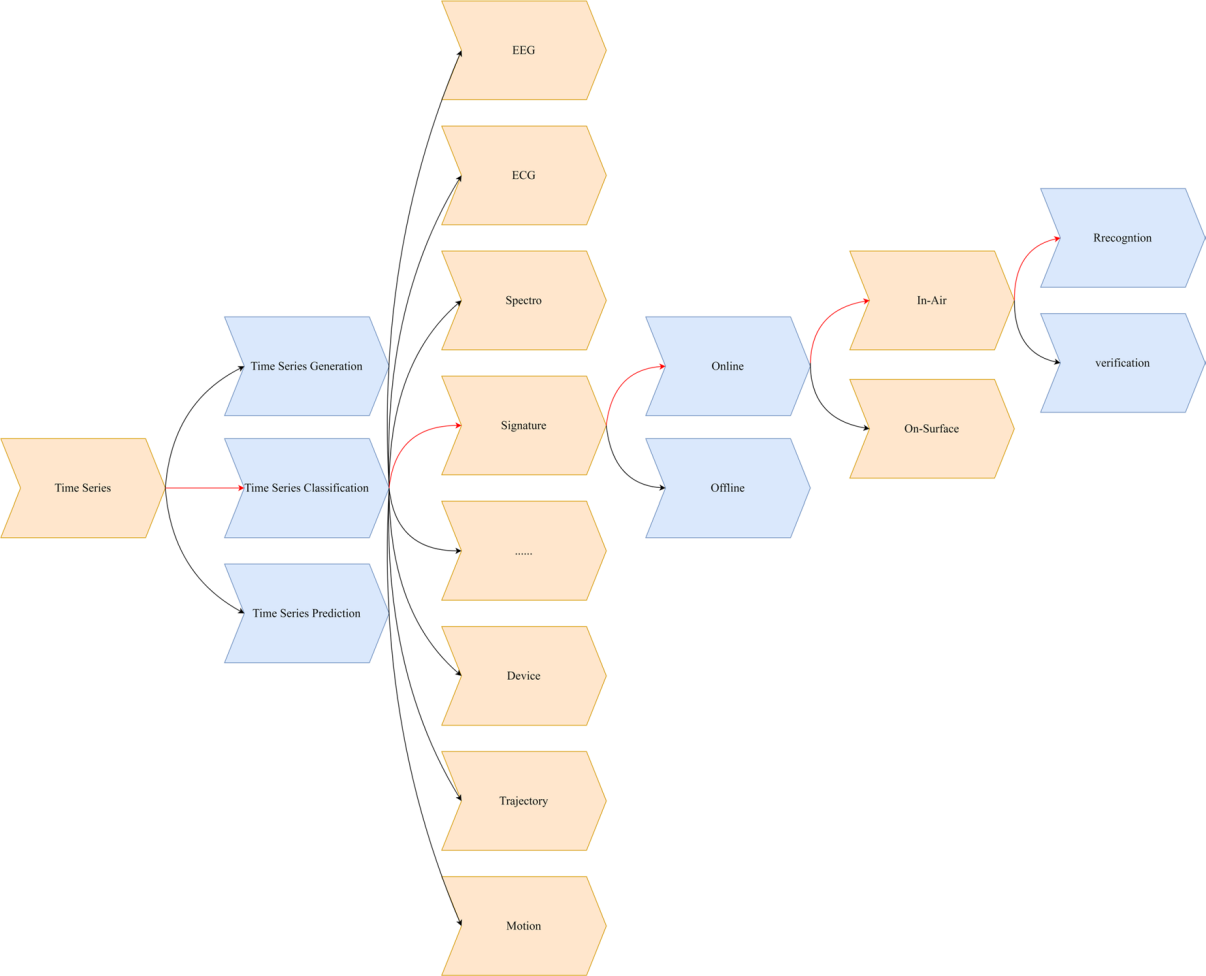
Research category of signature analysis. Signature analysis contains subcategories of online signature analysis, offline signature analysis, while the online signature analysis contain subcategory of on-surface signature analysis and in-air signature analysis.

In this section, we will cover some of the recent research progress in multiple areas of signature analysis, as shown in Fig. 2, including offline signature-related, on-surface signature-related, and in-air signatures-related research.

### Offline signature

Offline signature studies are generally related to the analysis conducted on static signature images^15^. Recent advancements in offline handwritten signature analysis utilize deep learning techniques for enhanced accuracy and reliability. Liu et al. introduced Mutual Signature DenseNet (MSDN), emphasizing detailed feature extraction from local signature regions and combining high and low-level features, validated on CEDAR^16^ and GPDS Synthetic datasets^17^^18^. Zheng et al. utilized max-pooling in CNNs to capture micro deformations in signatures, distinguishing genuine from forgery signatures across GPDS synthetic, CEDAR, UTSig^19^, and BHSig260 datasets^20^ using SVM classifiers^21^. Parcham et al. proposed CBCapsNet, a CNN-Capsule hybrid model for writer-independent verification, addressing traditional CNN limitations^22^. Banerjee et al. developed a BRDA-based feature selection method using SVD-transformed signals to enhance language-invariant offline signature verification^23^. Suehiro et al. introduced PCF, a Siamese network approach for reliable verification through pairwise signature comparisons and advanced learning techniques^15^. Longjam et al. proposed a CNN-BiLSTM model for writer-independent verification across multiple datasets^24^, while Tsourounis et al. enhanced feature learning with pre-training and contrastive loss^25^. These advancements illustrate the evolving landscape of deep learning methods in improving offline signature analysis systems.

### Online signature

In this subsection, we will discuss the recent representative progress for online signature analysis, including the on-surface and in-air signatures research areas as shown in Fig. 2.

#### On-surface signature

On-surface signature, as a subcategory of online signature, refers to signature performed on digital surfaces with time-variant dynamic information recorded^1^. Recent progress in on-surface signature analysis has introduced diverse methodologies to enhance accuracy and address various challenges. Lai et al. utilize the Sigma Lognormal model^26^ to synthesize signatures with varied distortions, improving verification against skilled forgeries^27^. Chandra et al. extract dynamic features from the SVC2004 dataset^28^, achieving 94% accuracy with Random Forest as a robust classifier^29^. Saleem et al. optimize signer-dependent sampling frequencies without interpolation, enhancing accuracy across datasets^30^. Vorugunti et al. introduce COMPOSV, a convolution-based framework that reduces features and improves fusion efficiency for diverse signers^31^. Diaz et al. simulate anthropomorphic features using function-based and histogram-based classifiers, significantly enhancing accuracy^32^. Faundez-Zanuy et al. propose biologically inspired feature splitting and classifier fusion, integrating pressure and spatial information effectively^33^. Faundez also explores the cognitive aspects of handwriting biometrics, highlighting the utility of the sigma-lognormal model^26^ in explaining human motor control^34^. Alonso et al. analyze online signature trajectories to distinguish healthy and pathological signatures^35^. Candela et al. detect cognitive impairment using multimodal data, achieving high accuracy with deep learning models^36^. Faundez et al. investigate gender differences in online handwriting signals using the BIOSECUR-ID dataset^37^^38^. Bhowal et al. propose a two-tier ensemble approach for writer-dependent verification, enhancing recognition accuracy^39^.

#### In-air signature

In-air signature refers to the contactless dynamic signature trajectories without the use of digital surfaces and typical in-air signature collection devices include mobile phone^14^, smartwatch^1^, camera^40^, and leap motion controller^41^ etc. Numerous models and methods have been proposed for in-air signature recognition. Gen Li and Hiroyuki Sato applied bidirectional LSTM to 22 subjects, obtaining a 0.83% EER^1^. Yeo et al.^42^ developed a faster version of DTW^43^ for more efficient in-air signature verification, with a 0.098 AER. Bailador et al.^44^ employed HMM^45^, DTW^43^, and a Bayes classifier to investigate imposter attacks on in-air signature patterns, thoroughly examining temporal changes. Malik et al.^2^ proposed a CNN based on hand-pose estimation with 1800 signatures, achieving a 67.6% improvement over DTW^43^. Buriro et al.^46^ utilized an MLP^6^ to classify 30 subjects, reaching an accuracy of 95%. Baljit et al.^47^ analyzed mobile phone users’ keystroke patterns using Random Forest^48^ and KNN^49^, achieving a 2.9% EER. Oğuz et al.^50^ used accelerometer signals from the in-air signature process to identify human activities via KNN^49^ and randomized neural networks^51^, achieving 99.994% and 99.97% accuracy, respectively. To fully utilize the ability of offline signature recognition models, as one of our previous works, we transform the online in-air signature into static information and then use multiple image recognition models for in-air signature recognition^52^. Meanwhile, synthetic 3D on-air signatures that mimic neuromotor control processes, offering new trajectories, kinematic data, and duplicates, validated through performance comparisons have been proposed and the generated databases can be used for research purposes^53^.

The comparative research between on-surface and in-air signatures suggests they contain an equal amount of information and more research for in-air signatures is necessary^35^. Nevertheless, with all the offline and on-surface signature datasets available^54^, there is rarely any in-air signature dataset that could be used for benchmarking. Therefore, in the next section, we will demonstrate our proposed multi-variate in-air signature dataset (MIAS-427) and illustrate its statistical properties.

### Limitations of previous approaches

Despite the significant progress in deep learning-based online signature recognition, several challenges remain:

- Data scarcity: Many deep learning models require large amounts of labeled data for training, which is often unavailable for online signature recognition.
- Device variability: Performance degradation is often observed when models trained on one device are applied to signatures collected from another device.
- Interpretability: Deep learning models are often criticized for their lack of interpretability, making it difficult to understand the underlying decision-making process.
- Computational complexity: State-of-the-art models like transformers and hybrid architectures are computationally expensive, limiting their use in real-time applications.

## Proposed online in-air signature dataset

In this section, we will discuss the data collection process, dataset statistical descriptions, and the dataset comparison among other datasets. In the dataset collection subsection, we introduce our in-air signature data collection application and discuss our data collection protocols. Meanwhile, we point out some of the limitations of our newly collected dataset such that researchers could use it with caution. In the dataset description section, we introduce the Dynamic time Warping^43^, and multi-domain descriptors to demonstrate some of the limitations of traditional algorithmic methods and ensemble learning approach^55^ when applied to in-air signature recognition, from which we could also present our dataset more statistically. In the dataset comparison part, we compare our dataset with other currently popular time series datasets and signature-related datasets respectively.

### Dataset collection

In this subsection, we discuss the data collection procedure and the details of the design of the in-air signature collection application.

During the online in-air signature collection phase, we collected in-air signatures from 427 participants, with the majority of them being college students, using mobile phone devices.Each participant was asked to sign their 9 dimensional in-air signature similar to how they would sign a handwritten signature in the air in 10 rounds. To eliminate the temporal memorization issue that generally exists in the in-air signature collection^44^, we followed strict 7 days separation, which means each participant performed 5 rounds of signature during the first section and another 5 rounds during the second section after 7 days, within each section we separate each in-air signature collection by a 10 minutes interval.

**Fig. 3:**
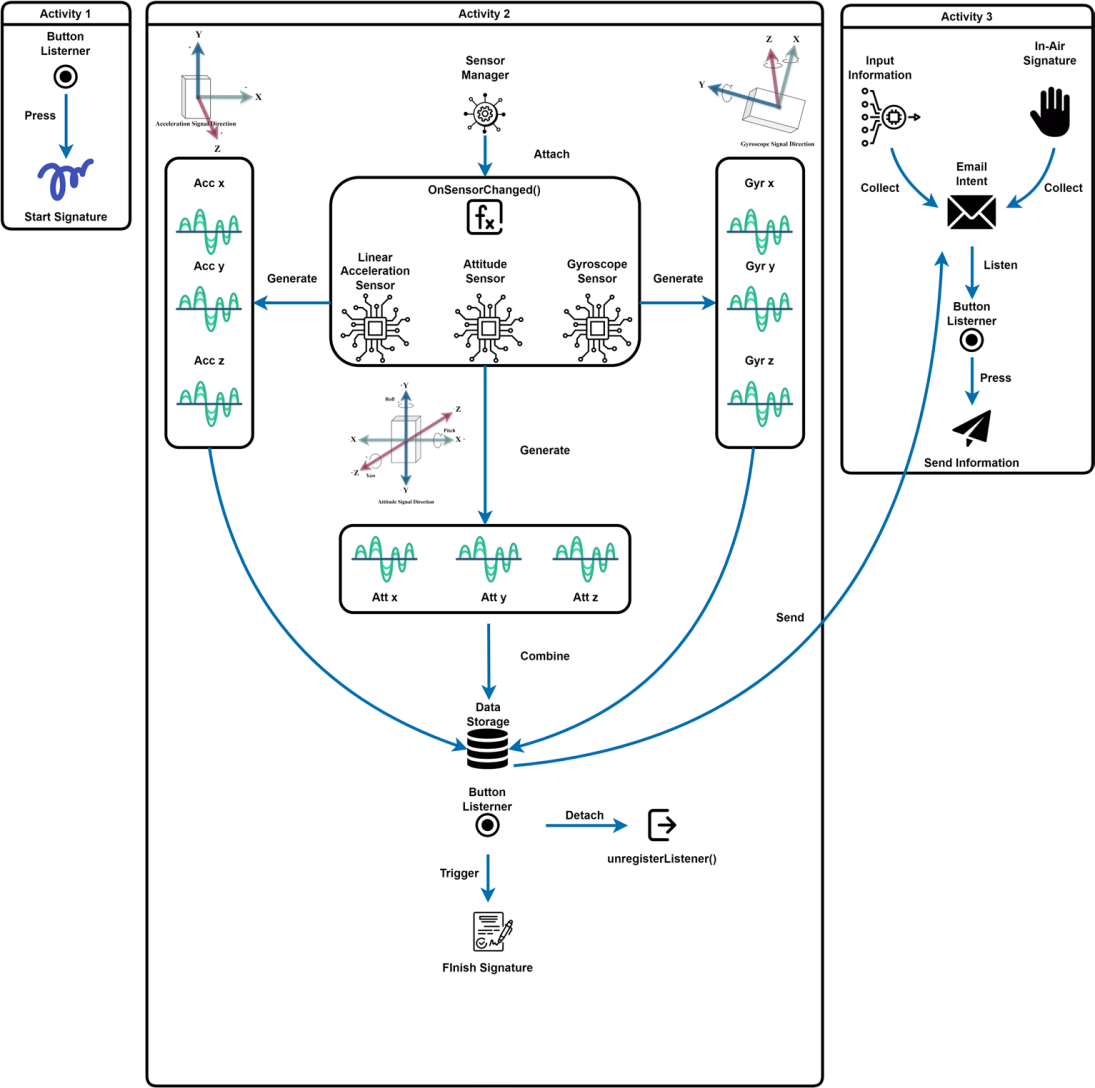
Online in-air signature data collection application design, in which 3 activities are described.

As shown in Fig. 3, we collect the online in-air signature from participants through our data collection application designed for mobile phones. The participants started the signature process by pushing the *start signature* button when they were ready and pushing the *finish signature* button upon completion. The temporary 9-dimensional in-air signature data was stored within the application. After finishing the signing process, participants were directed to a final application page where they entered their name, and the index of signature rounds, and selected their session. Once the information was submitted, the temporarily stored data was automatically sent to a designated email address for our later analysis.

There are 3 activities happening in the data collection applications that get involved during the in-air signature signing process as shown in Fig. 3. The first activity displays a guidance page with relevant information about our research introduction, the in-air signature collection process, and notifications, and includes a button to start the in-air signature signing process. When this button is pressed, the signature collection begins, and the application transits to the second activity. Here, the sensor manager acts as the core for the accelerometer sensor, gyroscope sensor, and attitude sensor. Once activated, all the sensors remain attached to the sensor manager throughout the second activity. The x, y, and z coordinates are recorded in a synchronized manner. The specific definition for the orientation of every sensor is depicted in Fig. 3, and the description of each dimension is presented in Table 1.

**Table 1:** Dimension description of the online in-air signature data.

Dimension description			
Index	Sensor	Description	Abbreviation
1	Accelerometer	x-axis	$acc_x$
2	Accelerometer	y-axis	$acc_y$
3	Accelerometer	z-axis	$acc_z$
4	Gyroscope	x-axis	$gyr_x$
5	Gyroscope	y-axis	$gyr_y$
6	Gryroscope	z-axis	$gyr_z$
7	Attitude	pitch-axis	$att_x$
8	Attitude	roll-axis	$att_y$
9	Attitude	yaw-axis	$att_z$

**Fig. 4:**
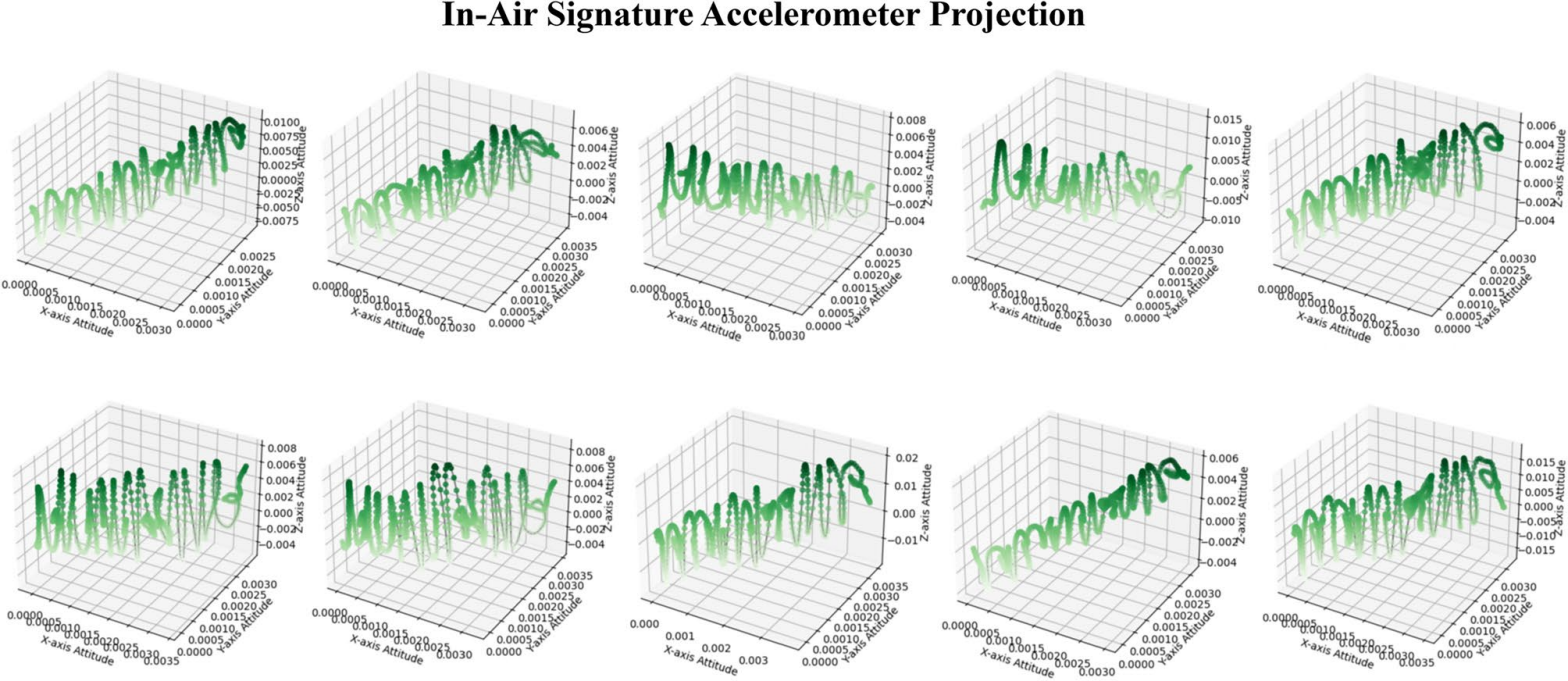
Ten rounds of online multi-variate in-air signature projection from the accelerometer perspective.

Here, we take the dimensions of the accelerometer as an example. For the accelerometer as shown in Fig. 1, the Android Studio axis coordinate system defines the positive x-axis direction as the horizontal line from the left to the right when holding the phone facing the participants, the positive z-axis direction as going out of the paper, and the positive y-axis direction is defined as pointing toward the user’s head. These coordinate directions remain constant regardless of the phone’s orientation as long as it is being held through the in-air signature process. In “Experiment”, under the data processing subsection, we will present the nine-dimensional features data format collected for a sample in-air signature for illustration. The unit for the sensor data is meters per second squared. Table 2 shows the sensor sampling frequency that can be adjusted using the *sensor_delay* parameter. The table lists the available sampling rates for the application with a couple of milliseconds variation for different sensors. In this experiment, considering the performance of the phone and battery, we used *sensor_delay_normal* for both the accelerometer sensor, gyroscope sensor, and attitude sensor, resulting in a sampling interval of around 215-230 ms per sample. The sensors handle sampling requests through the *OnSensorChanged()* function. After the participant completes a signature and presses the *finish signature* button, all sensors are detached from the sensor manager to save the battery and improve the software performance. Participants are then directed to the third activity, where they enter their names and the number of rounds. This third activity includes an email intent object that retrieves the temporarily stored data and automatically sends it to the designated email address. As shown in Fig. 4, we demonstrate a sample with 10 rounds of the general contour of our newly collected online in-air signature data. As the in-air signature is time series data of nine dimensions, the specific trajectory shape of the in-air signature could not be provided by three sensors, here we illustrate their three-dimensional projection from the accelerometer perspective. The x, y, and z axes are the 3 dimensions of the accelerometer, with a deeper green color representing a later timestamp and vice versa.

**Table 2:** Available sampling parameters and sampling rate for Android Studio SDK.

Available sampling rate	
Parameter	Sampling rate (ms)
FASTEST	18–20
GAME	37–39
UI	85–87
NORMAL	215–230

#### Challenges and potential biases in the dataset

The aforementioned procedure concludes our data collection process. Nevertheless, we need to point out several shortcomings in our data collection phase so that researchers can use it with caution.

- Large dataset collection is a complicated process that involves participant organization, guidance deceleration, agreement content signing, etc. Unfortunately, we lost several in-air signature data during the data transfer, which is listed in Table 3. In our experiment, we compensate for this part of the data by re-weighting the frequency of their appearance in the training and testing sets.
- Based on our observation, during the in-air signature collection phase, participants will generally star at the phone for a couple of seconds after they press the *start* button and star at the phone again to press *finish*. Inevitably, there are two short noisy periods at the beginning and the ending parts of the in-air signature signal. We did not exclude those two parts for the sack of completeness, but we will demonstrate in “Experiment” through the CAM^56^ of the in-air signature from a couple of participants and discuss how those two periods also, sometimes, contribute to the in-air signature recognition.
- Participants demographics indicates that the majority of the participants are college students aging form 19 to 22 years-old, which means the MIAS-427 might be biased when applied to senior populations. In the future, an in-air signature dataset that covers a broader variety of population should be collected to eliminate the potential bias for the dataset.

#### Impact and limitation of temporal separation

We followed a separation procedure of data collection to avoid temporal memorization. Even though this separation could eliminate the temporal memorization of the in-air signature patterns to some degree, the in-air signature is highly user-dependent and some participant’s signature patterns could vary after 20 days or even longer as indicated by some research^44^. Limited to time and resources, chronic research dedicated to the long-term in-air signature pattern variance is necessary and we list this in “Experiment” for the future. Here, we discuss the possible impact and limitation introduced by this methodology:

- Recognition performance: temporal separation can improve recognition performance by capturing more natural variations in signature patterns over time. This is particularly important for real-world applications, where signatures may be collected days or weeks apart.
- User variability: while the 7-day interval reduces memorization effects, it may also introduce variability due to changes in the participant’s motoric behavior over time. For example, factors such as mood, physical condition, or environmental context could influence signature patterns.
- Data consistency: the temporal separation protocol requires careful scheduling and coordination with participants, which can be challenging in large-scale studies. Additionally, participants may drop out or fail to complete the second session, leading to incomplete data.

**Table 3:** The missing online in-air signature data index.

Missing data																							
Participant index	16	18	18	58	78	88	99	100	137	156	165	178	189	204	210	211	230	239	270	270	278	279	280
Signature index	5	6	7	4	3	4	8	1	7	9	8	5	3	7	4	5	5	6	6	9	4	2	1
Section	1	2	2	1	1	1	2	1	2	2	2	1	1	2	1	1	1	2	2	2	1	1	1

####  Data regulations

We provide the following statement regarding data collection and participant consent. The dataset was primarily collected from students at Dongying Vocational Institute and Shandong Institute of Petrochemical Technology. The Dongying Vocational Institute and Shandong Institute of Petrochemical Technology approved the experiment protocols and all relevant details, including the participant in-air signature collection process and potential research purpose for this dataset. All methods were performed in accordance with the relevant guidelines and regulations. This study did not require additional ethics approval, as it involved no clinical trials with humans or animals and adhered to ethical standards. All participants, who were adults participating voluntarily, provided informed consent prior to involvement and were free to choose whether to participate. Anonymity and confidentiality were strictly maintained, and participation was fully voluntary. The study relied exclusively on high-dimensional, anonymized in-air signature data, free from personally identifiable information. Participants retain the right to withdraw their data from the study at any time by notifying the data collector, upon which all relevant signature data will be securely deleted. The experiment were performed in accordance with relevant guidelines and regulations and complies with General Data Protection Regulation (GDPR) standards. Before data collection, all participants were informed of the study’s purpose and the intended use of their data. In recognition of their time and effort, participants were fairly compensated, and all data were stored securely without personal identifiers.

### Dataset description

**Fig. 5:**
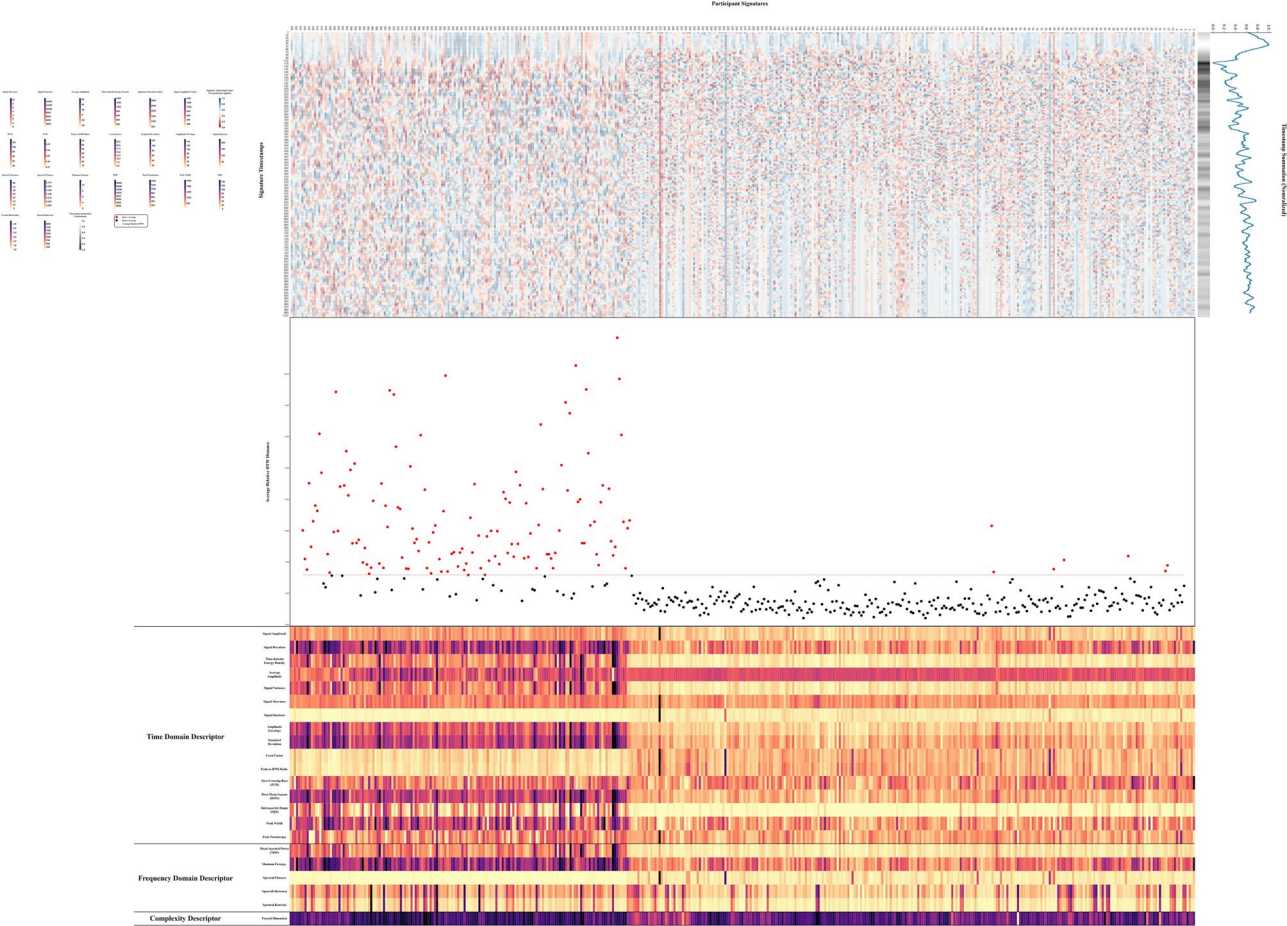
Heatmap Description of the new mobile phone online in-air signature dataset (MIAS-427) with relative dynamic time warping distances. Time, frequency, and complexity domain descriptors are provided for better illustration.

This subsection serves two purposes:

- The implementation of timestamp heatmap, relative dynamic time warping algorithms^43^, and multiple domain descriptors serve as the auxiliary statistical properties illustration of our MIAS-427 dataset.
- Meanwhile, we demonstrate the limitation of DTW and ensemble learning for in-air signatures recognition, we use Fig. 5 to explain why we finally choose the deep learning-related models for the model selection in “Experiment”.

As discussed in “Literature review”, we demonstrate the recent research direction based on the signature completion agent. Nevertheless, if we categorize the signature research based on the methodologies, there are generally three main approaches: Dynamic Time Warping-related^43^, deep learning related, and ensemble learning^55^.

The Fig. 5 contains three subplots. The scale color panel for the heatmap is represented at the top left corner of the chart. The top part is the statistical heatmap illustration of MIAS-427. The middle part is the average relative DTW distance, which summarizes the relative average distance among the in-air signature patterns of each participant. The bottom part is the 3 domain descriptors that are used to illustrate the statistical properties of our dataset.

Even though we will perform a thorough model selection process for in-air signature recognition, limited to time and resources, we will first rule out some of the approaches. The visualization in this section could help us empirically reveal some of the clear drawbacks of DTW and ensemble learning approaches based on multiple domain descriptors when applied to in-air signature recognition, which provides support for our use of deep learning models for model selection in “Experiment”.

#### Timestamp heatmap

In this part, we will first present and discuss the MIAS-427 dataset in a more statistical way using the timestamp heatmap.

The top subplot is a heatmap that summarizes the first 1000 time stamps of the participants’ in-air signatures. One thing to notice is the x coordinate for this heatmap, starting from the right to the left is the index of the subjects. As we explained in the previous subsection, each subject has 10 signature repetitions and for each repetition, we have three sensors, and for each sensor, we have 3 dimensions as shown in Table 1. To extract the main information, we take the average for each dimension, and each sensor, and merge them into only one time series that represent each unique subject. The color scale represents the intensity of the in-air signature values at each timestamp. By examining the heatmap from the right to the left, we can see how the in-air signature patterns vary among different subjects. The heatmap has alternating intensities, which demonstrates repetitive patterns in the signatures across subjects. Consistent patterns across several subjects indicate similarities in signature behavior, while areas with high variation represent differences in individual signature characteristics, which provides us with the supportive statistical basis for the later machine learning recognition models. The heatmap also suggests in-air signature participants outliers, including the participants with unexpectedly high or low values compared to the other participants, which demonstrates unique signature behaviors in the data collection. As we could observe, participants index *66*, *91*, *177*, *252* exhibit consistent high signal through the timestamps, while index *68*, *102*, *221* shows low signal values. Being able to visualize signature signals directly could help us and other researchers perform more user-targeted methodologies. Additionally, on the top-right part of the graph, we have the normalized time stamp values across each subject, which provide us with information to identify timestamps that are particularly significant or of common intensities. We could observe that the in-air signature generally starts with a heavy stroke and gradually switches to lighter trajectory patterns.

#### Average relative dynamic time warping (DTW)

This section will discuss the theory of the Dynamic Time Warping (DTW) algorithm and its shortcomings when applied to in-air signatures.

The middle subplot of Fig. 5 shows the relative average DTW distance of all participant’s signatures. DTW^43^ is a well-established algorithmic method used in time series analysis to measure the similarity between two temporal sequences. As suggested by some research, DTW is particularly effective in distinguishing different online signatures by calculating the distance between any pair of signals^57^. Algorithm 1 details the DTW procedure for comparing two sequential signals. It involves constructing a dynamic time-warping cost matrix with dimensions matching those of the input signals, which in this context are two sequential in-air signatures. Initialization of the cost matrix involves four conditions: the origin is set to zero, and the second elements of the second row and second column are initialized with the starting distance of each signal. Elements in the first row and first column are set to infinity. Progressing from the origin, each cell in the DTW cost matrix is assigned the minimum value from the neighboring four cells, adjusted with tuning coefficients. The final DTW distance is found in the last cell of the cost matrix. Tracing back from this cell to the origin, the warping path is determined by connecting the minimum value cells, thus identifying the shortest path to the origin. This warping path represents the alignment between the points of the two sequential signals. Unlike traditional point-by-point alignment, multiple equal elements in the horizontal or vertical directions of the cost matrix indicate a one-to-many alignment between the two signals.

**Algorithm 1:**
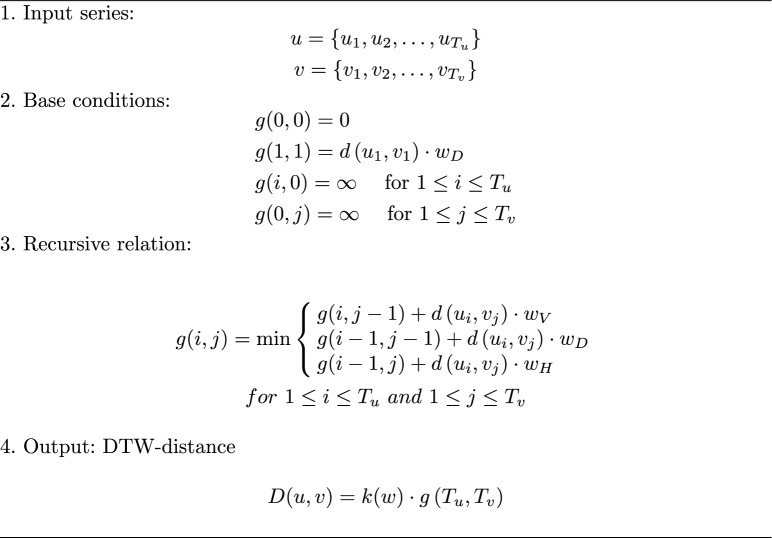
Dynamic time warping algorithm^58^.

**Fig. 6:**
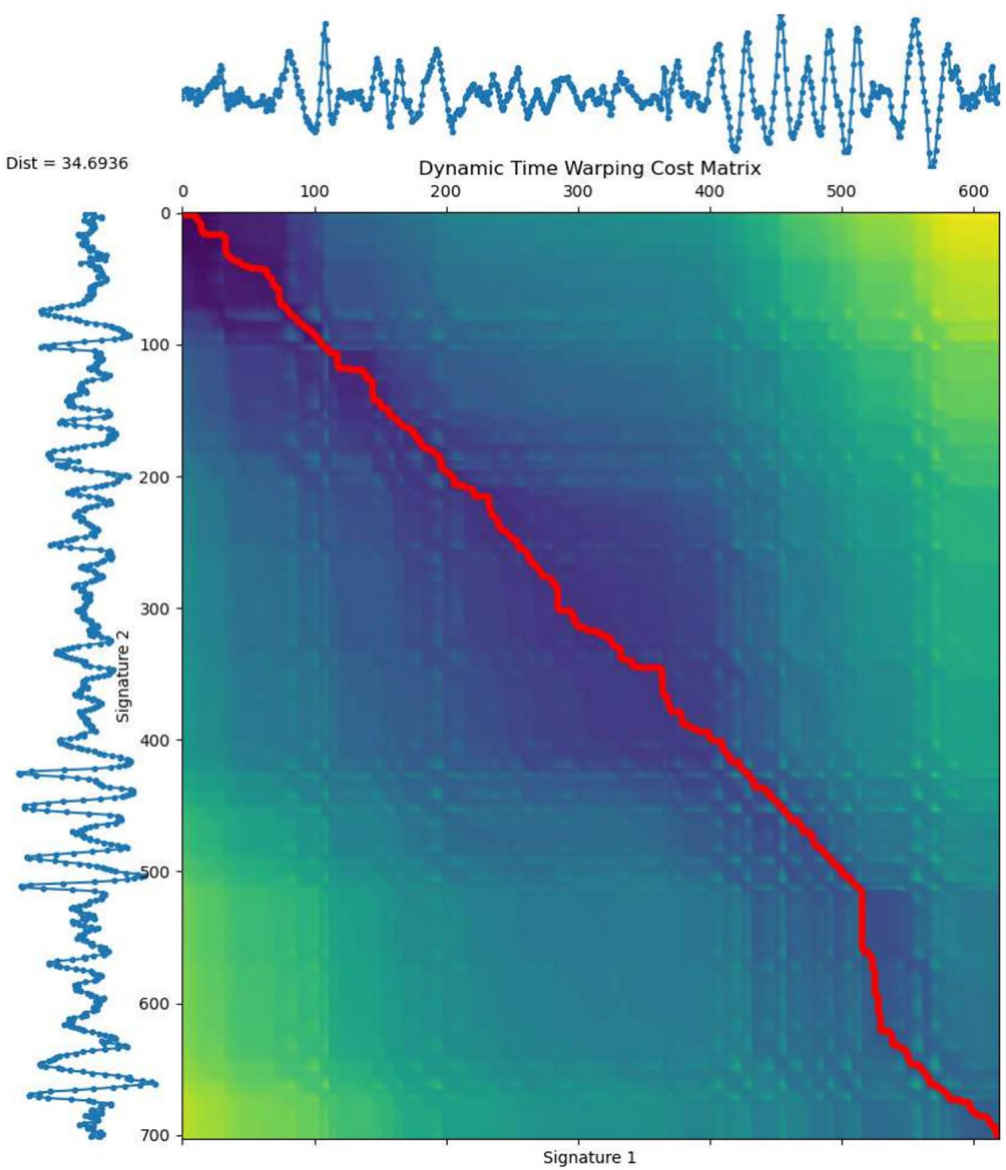
Dynamic time warping cost matrix between two in-air signatures of the same category.

The image in Fig. 6 illustrates a sample of the dynamic time warping (DTW) cost matrix. In this representation, two signature signals are depicted on the left and top of the graph, respectively, while the sample signals belong to the same signature category. The top-left corner of the matrix displays the DTW distance value for these two sample signals. According to the DTW algorithm, the element cost is color-coded from blue (lower cost) to light yellow (higher cost). The red line represents the warping path of the DTW cost matrix. When signals match perfectly, the warping path is a straight line from the origin to the last cell of the matrix. Blue areas around the warping path indicate lower alignment costs, whereas yellow areas, located farther from the warping path, indicate higher costs.

In this study, we use the aforementioned DTW algorithm to calculate the relative DTW distance for each participant’s signature signal by taking the average DTW of the current participant’s signature with all other signals. The resulting middle subplot in Fig. 5 features a red line representing the average relative DTW distance across all subjects. Vertically, each heatmap line corresponds to a specific subject’s relative average DTW distance, with red dots indicating DTW distances above the average and black dots indicating distances below the average.

As we could observe in the middle subplot of Fig. 5, within 2D feature spaces, the DTW algorithm could not provide efficient classification ability for in-air signatures. Using deep learning models to perform thorough experiments for a very large time series dataset is generally considered time-consuming and a large amount of computation resources are required^3^. Nevertheless, the nuanced differences in DTW distances observed between most participants’ in-air signatures suggest the necessity of employing more advanced methods for in-air signature recognition analysis. As indicated by some research, DTW, as an old-fashioned traditional method, has its limitations when applied to time series in-air signature recognition and verification tasks^1^. The limitation of traditional algorithmic methods like DTW serves as a strong incentive for us to turn our attention to more sophisticated deep learning models that will be discussed in “Experiment”, in which we thoroughly introduce the deep learning models that will be used in model selection and dimension-wise Shapley Value analysis procedures.

Additionally, an interesting phenomenon emerges around the 268th subject. As illustrated in Fig. 5, subjects before the 268th exhibit relative DTW distances predominantly below the average and centralized around similar relative DTW distances, whereas subjects after the 268th show distances largely above the average and tends to be more scattered apart. Upon review, the relative average DTW distance division around the 268th subject coincides with a change in the source of the data: the first part of the dataset is collected from the students of one college, while the second part is collected from another. Given the data collection conditions are maintained exactly the same, geographically close, this discrepancy in the relative DTW distances between the two data sources suggests a significant difference in signature pattern for their respective signals. This certainly adds difficulties to our in-air signature recognition task and, meanwhile, it reveals the fact that the in-air signature is highly user-targeted and it may be influenced by past personal experiences and collective cognition. Therefore, conducting dimension-wise feature selection for in-air signatures could help us form a more concrete foundation in understanding human cognition preferences when dealing with complicated cognition tasks like in-air signatures and pave the path for the future cognition computation for mimicking human cognitive process, which will be discussed more in detail in “Embodied cognition inspired feature selection”. Nevertheless, the exact cause of this difference is not immediately clear and requires further investigation and we list this phenomenon in “Research limitations and future work” for future research.

#### Domain descriptors

In this part, we introduce the idea behind ensemble learning and explain some of the limitations of this methodology when applied to in-air signature recognition.

Ensemble approaches are widely utilized in the field of online signature recognition, leveraging the combined results of multiple classifiers from various domains, with majority voting often used to determine the signature category^39^. A prominent example in time series recognition is FLAT-COTE, an ensemble model consisting of 35 classifiers from different domains, where each classifier contributes a weighted vote, and the class with the highest cumulative weight is selected as the prediction^59^. FLAT-COTE has demonstrated superior performance compared to traditional single models and even some convolutional neural networks (CNNs)^59^. Despite these successes, our analysis of descriptors within the time, frequency, and complexity domains, as illustrated in the lower subplot of Fig. 5, reveals that these descriptors effectively differentiate between two data source groups but fail to distinctly classify individual subjects. This ambiguity among multiple descriptors suggests that traditional ensemble classifiers based on multiple domains may not achieve high accuracy in recognizing in-air signatures. Given this limitation, our research focuses on exploring various end-to-end deep-learning models for in-air signature recognition. In “Experiment”, we conduct a comprehensive model selection process to identify the most suitable model for this task, aiming to enhance recognition accuracy and reliability beyond what traditional ensemble methods can offer.

Therefore, we finally choose 7 end-to-end deep learning models from the popular time series model candidates^3^ for the model selection process detailed in “Experiment” to achieve better precision for in-air signature recognition.

### Dataset comparison

**Fig. 7:**
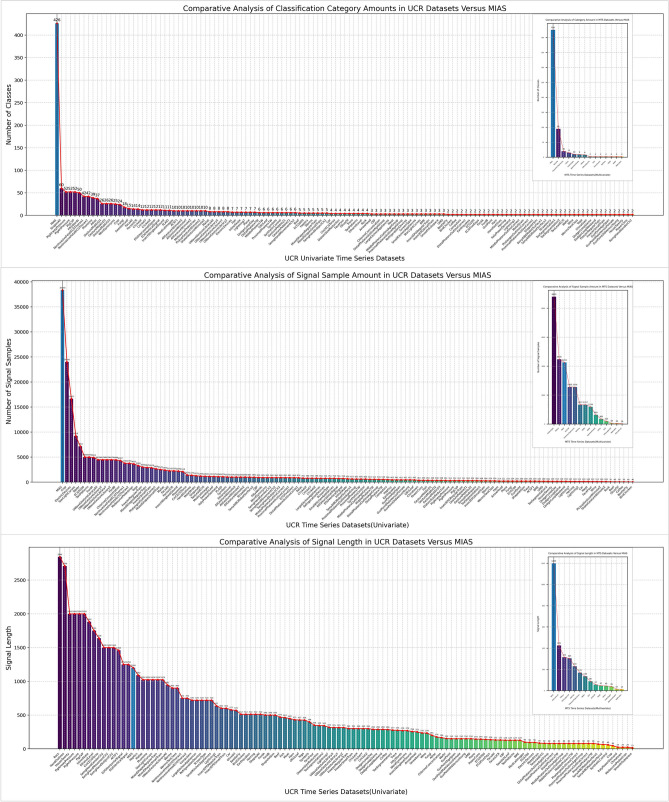
Dataset comparison between our newly proposed MIAS-427 and UCR^60^ and MTS^61^ (top-right corner) in terms of number of classification categories, number of samples, and signal length.

To better visualize how the MIAS-427 dataset is considered advantageous compared with other time series datasets, here we list three subplots in one chart as shown in Fig. 7, in which we demonstrate MIAS-427’s advantage from three perspectives, including the classification category, signal sample amounts, and signal length. The datasets we compare with are the UCR uni-variate dataset collection^60^ and MTS dataset collection^61^. The UCR Time Series Classification Archive is a comprehensive repository of time series datasets spanning various research fields^60^. It serves as a benchmarking resource for evaluating the performance of time series classification algorithms and is considered to be one of the largest time series dataset collections^3^. The UCR contains uni-variate datasets across 16 time series research areas. While the MTS dataset collection contains a set of time series datasets of multi-dimensions. The plot on the top right corner of each subplot in Fig. 7 is the comparison between the MIAS-427 and the rest of the multi-variate MTS dataset. Starting from the first subplot is the classification category amounts comparison with the UCR dataset collection. We have 427 classification categories, which is 7 times bigger than the largest dataset in the UCR dataset collection, and it’s also much bigger compared to every single one of the multi-variate datasets in MTS in the perspective of the number of categories. The second chart is the comparison in terms of the signal sample amount, the MIAS-427 dataset is also the biggest compared with the rest of the uni-variate datasets in the UCR, but it comes to the third position if compared with the MTS datasets collection in this perspective. For the third graph, in terms of the signal length, even though, MIAS-427 is not the dataset that contains the longest signal samples, it’s certainly in the top-tier position for the uni-variate signal dataset. It’s worth noting that the signal length is the longest in terms of the multi-variate dataset compared with the others in MTS. Therefore, the MIAS-427 dataset could be considered one of the largest in the field of time series datasets from various perspectives.

In the field of signature analysis, there are several offline signature datasets, such as CEDAR^16^, GPDS^17^, UTSig^19^ and BHSig^20^. Additionally, there are on-surface signature datasets like DeepSignDB^62^, SVC^28^ and MCYT^63^. For in-air signatures, besides MIAS-427, the dataset proposed by Behera et al. is considered a large in-air signature corpus collected using Leap Motion Controllers^41^. Nevertheless, there is still a scarcity of datasets for in-air signatures, and more resources are needed to advance in-air signature analysis.

### Real-world applications of MIAS-427

The MIAS-427 dataset has significant applications in security, digital authentication, and biometrics:

- Biometric authentication: Enables testings for secure access control, multi-factor authentication (MFA), and fraud detection in identity verification systems.
- Digital signatures: Supports the testing for secure document signing in financial and legal transactions, as well as blockchain-based identity verification.
- Contact-less authentication: Facilitates gesture-based unlocking for smartphones and smartwatches, enhancing security in mobile devices.
- AI-powered fraud prevention: Helps develop adaptive security models that detect anomalies in signing behavior and prevent identity spoofing.

These applications demonstrate MIAS-427’s potential in enhancing authentication and security across various domains.

## Time-series deep learning model structures

The purpose of this section is to briefly discuss the models used for the in-air signature recognition model selection procedure. The best-selected model for mobile phones (MIAS-427) and smartwatches^1^ will be used for dimension-wise Shapley Value analysis in “Experiment”.

In this section, following the time series journal review by Ismail Fawaz et al.^3^, which is a comprehensive end-to-end deep learning model evaluation on UCR/UEA (uni-variate)^60^ and MTS (multi-variate)^61^ time series dataset. We chose 7 models for our model selection and 6 of them are chosen from this review journal article to apply to both the wrist-worn smartwatch dataset^1^ and our newly collected hand-held mobile phone online in-air signature dataset, including MLP^4,6^, Time-CNN^11^, MC-DCNN^9^, Encoder^8^, FCN^4^, and ResNet^7^. Additionally, we also use InceptionTime^12^ as it’s one of the recently proposed models that are designed specifically for time-series classification. The model structures figures and dimensions shown in this section are tailored for the first smartwatch data^1^ as in our previous work^13^ to show as a sample, and only one dimension signal of the signature is illustrated (except MC-DCNN), while the specific parameters of our newly collect mobile phone in-air signature data (MIAS-427) are tested under the same model structures but revised accordingly to make sure that two datasets are under the same experimental conditions.

### Multi-layer perceptron (MLP)

**Fig. 8:**
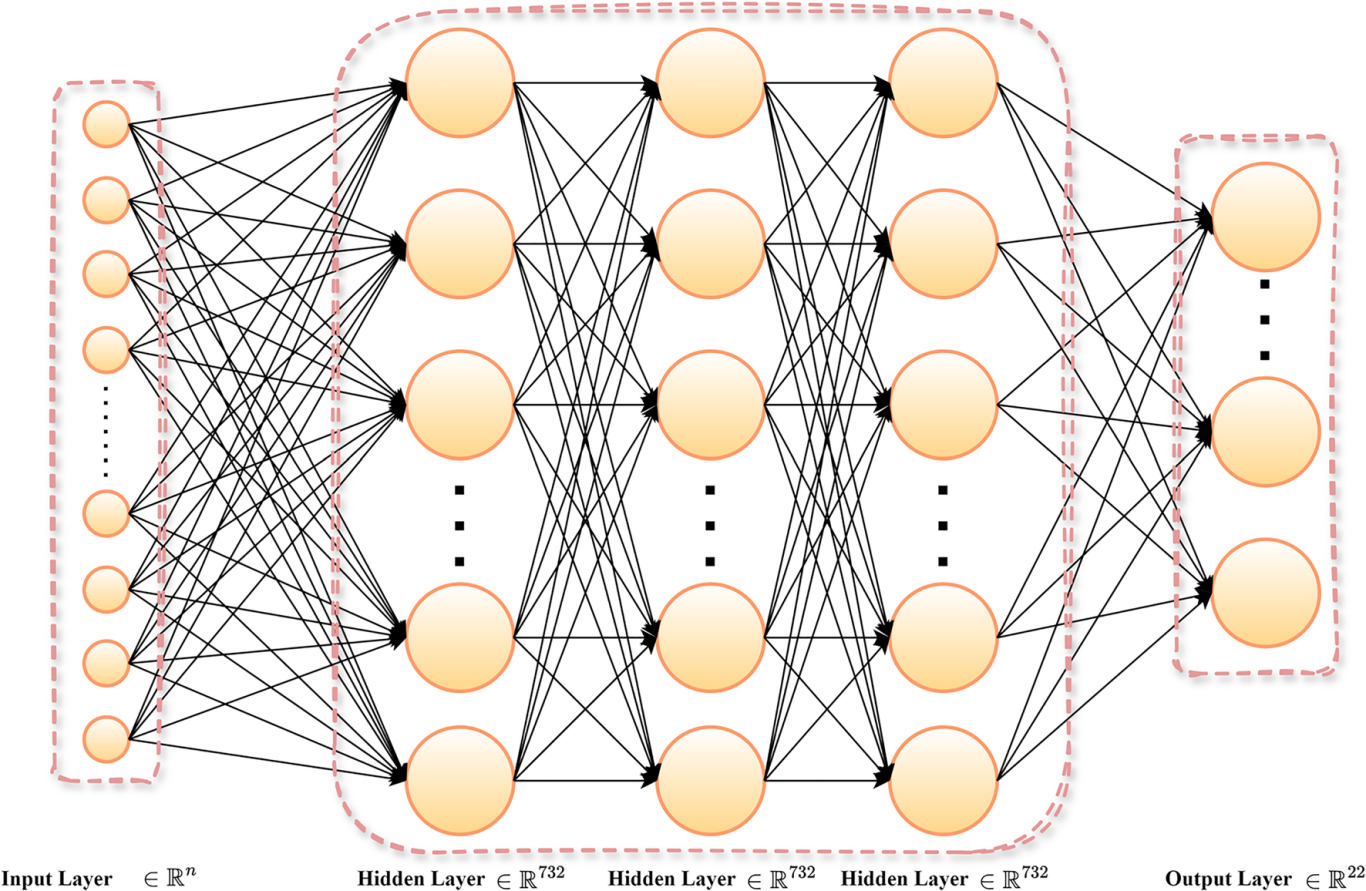
Time-series multi-layer perceptron model structure.

We choose multilayer perceptron (MLP)^6^ as the first model in our model selection procedure discussed later in “Experiment” and introduce it here as it is one of the earliest machine learning models proposed and it has been applied to various field, particularly in the signature analysis^64^. The input dimension is adjusted to the signature dimension flattened for time series classification if multi-variate signals are considered^3,4^. The architecture is straightforward, as depicted in Fig. 8 represents the MLP structure used in our experiment, featuring one input layer, three hidden layers, and one output layer. With the option to adjust this number for fine-tuning purposes, each hidden layer consists of 732 neurons in our experiment. Several key components are worth noting, including the weights, biases, and activation function. The output layer utilizes a SoftMax^65^ and the number of output neurons corresponds to the number of subjects in the experiment.

### Time-CNN

**Fig. 9:**
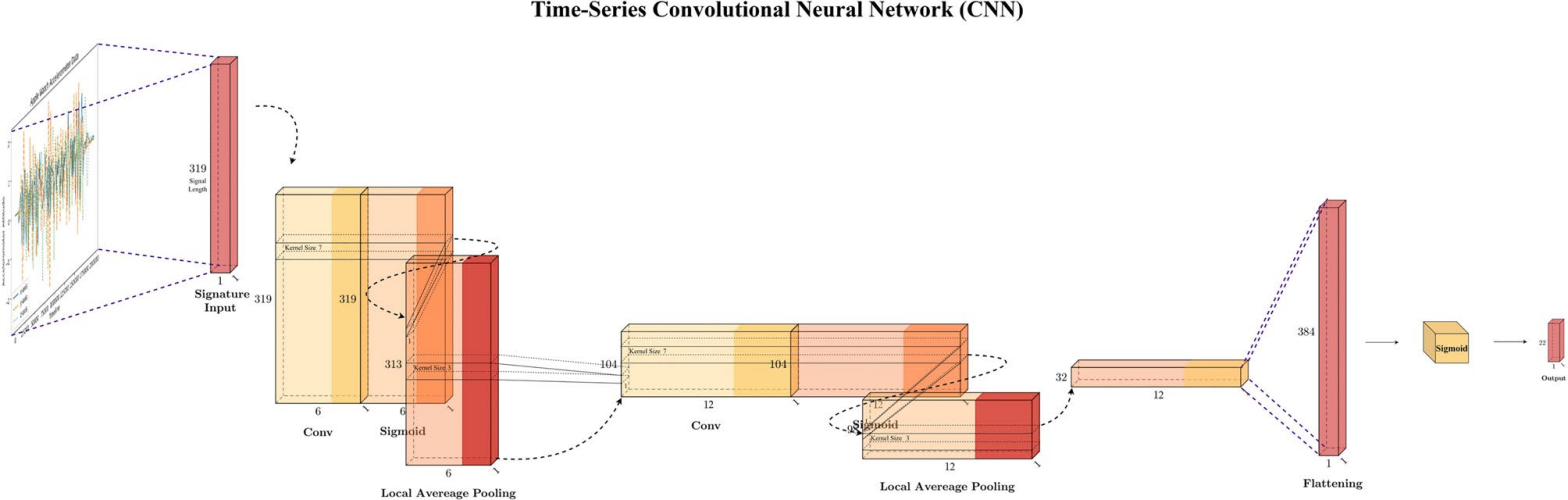
Time-series time-CNN model structure.

The subsequent model investigated in the experiment is the Time-CNN^11^, depicted in Fig. 9, as the first CNN-related model discussed here, its structure is relatively simple compared with the other model that we will be covering later. Time-CNN is a variation that’s designed for the time series classification^3^, comprising two convolutional layers and two average-pooling layers. The average pooling layers have a stride of 3 and a kernel size of 3. In the convolutional layer, the number of filters is of kernel size of 7 with 6 and 12 filters respectively. Ultimately, the feature vectors from the convolutional layer are concatenated into a flattened layer and classified into the number of subjects using softmax activation.

### Multi-channel deep convolutional neural network (MC-DCNN)

**Fig. 10:**
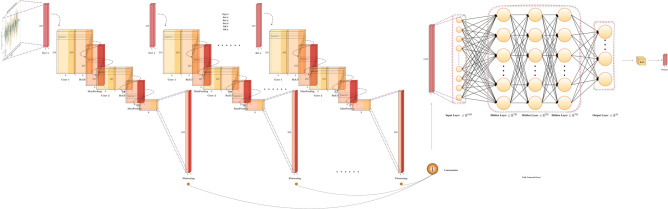
Time-series MC-DCNN model structure.

The next model under consideration is the Multi-Channel Deep Convolutional Neural Network (MC-DCNN)^9^, which is a network designed specifically for time-series classification of multivariate data information, and it has been used for the detection of general human activities^66^ and manufacturing^67^. As depicted in Fig. 10, a single channel comprises 2 convolutional layers and max-pooling layers respectively. Each convolutional layer employs a kernel size of 5 with 8 filters, while the max-pooling layers following convolution have both stride and kernel size set to 2. Subsequently, after processing each channel, the resulting feature vectors are concatenated and serve as the input for the fully connected layers. The hidden layer of the fully connected layer consists of 732 neurons, with the output layer configured for the number of subjects represented in the in-air signature dataset. What sets this model apart from other CNN architectures is its utilization of multi-channel separation. Considering the 1-D nature of signal processing, for other models, our dataset input would be [$Signature_{Length}, Signature_{Dimension}$]. Nevertheless, for MC-DCNN, each dimension of the in-air signature time series undergoes separation, resulting in a total of 9 channels for the dataset. Within each channel, data convolution is conducted independently. Similarly, ReLU^68^ activation is employed for each channel’s convolution operation, while SoftMax^65^ is utilized for classification purposes.

### CNN+attention

**Fig. 11:**
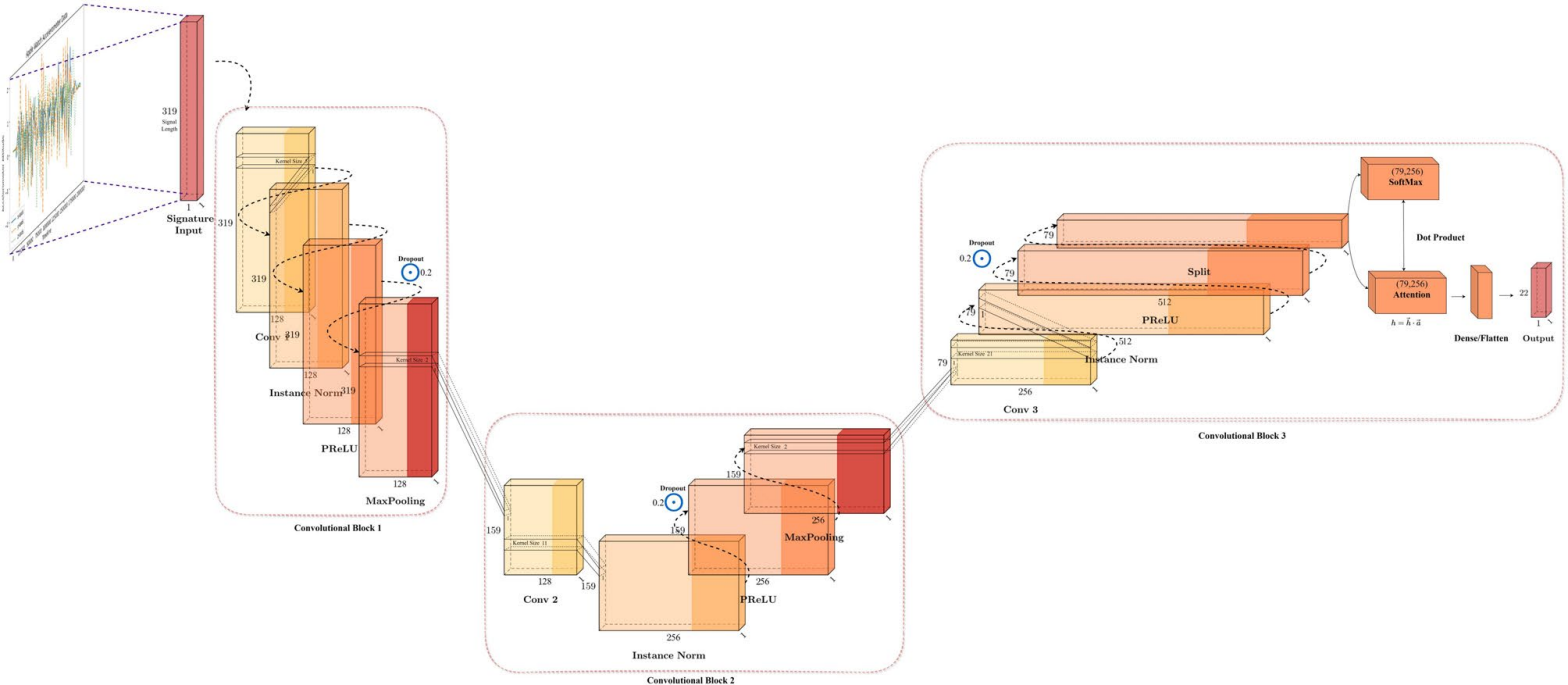
Time-series encoder model structure.

The next model evaluated is an hybrid CNN and Attention structure^3^. Attention mechanisms are widely used in deep learning, especially in the context of sequence modeling and natural language processing^69^. We include this model for our in-air signature recognition model selection in “Experiment”. As illustrated in Fig. 11, unlike the earlier models, to learn the slope coefficients for each convolution, this model uses Parametric Rectified Linear Unit (PReLU)^70^ and employs instance normalization^71^ to normalize across the entire sample. A notable difference lies in the attention mechanism implemented in the final layer. After the last convolution, the time series data of in-air signatures is divided into two segments. One of these segments goes through the SoftMax function^65^ to serve as attention or weighting for the other segment. Subsequently, these segments are merged using a dot product, a departure from the self-attention mechanisms typically seen in transformers^72^. The model comprises 3 convolutional layers, with two of them followed by a max-pooling layer. Both max-pooling layers have a stride and kernel size of 2. The convolutional layers maintain consistent stride and padding values of 1 and 0, respectively, with kernel sizes of [5, 11, 21] and corresponding filter sizes of [128, 256, 512]^3^. Dropout is also utilized to prevent overfitting.

### Fully convolutional network (FCN)

**Fig. 12:**
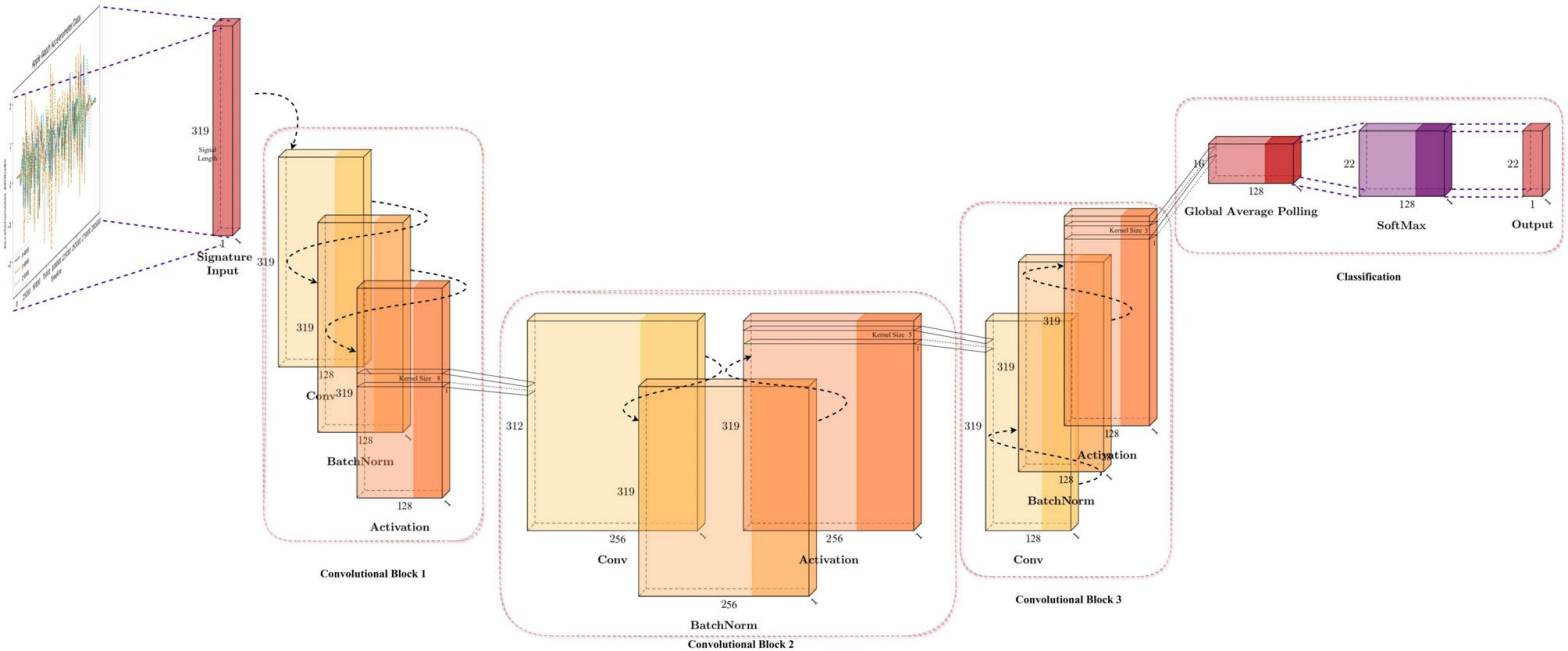
Time-Series Fully Convolutional Network Model structure.

Fully Convolutional Network (FCN) is a deep learning structure originally used for image segmentation^5^. It also shows outstanding performance in time series classification^4^ and enables the effective capture of temporal patterns and dependencies in sequential data^73^. Unlike the traditional FCN with $1\times 1$ convolutional layer and transpose convolution attached at the end, a replacement of the global average pooling layer is used for the time series online signature recognition. As depicted in Fig. 12, the network consists of one global average pooling layer and three convolutional layers. The convolutional layers are of kernel sizes 8, 5, and 3 with 128, 256, and 128 filters respectively. Each layer uses batch normalization^74^ and ReLU^68^ functions. Both the padding and stride for these layers are consistently set to 0 and 1. The global average pooling layer facilitates the generation of class activation maps^56^, which can help us identify the most distinguishable features within time series in-air signature signals^3,4^. Finally, a SoftMax^65^ classifier categorizes the different signatures into specific subjects.

### ResNet

**Fig. 13:**
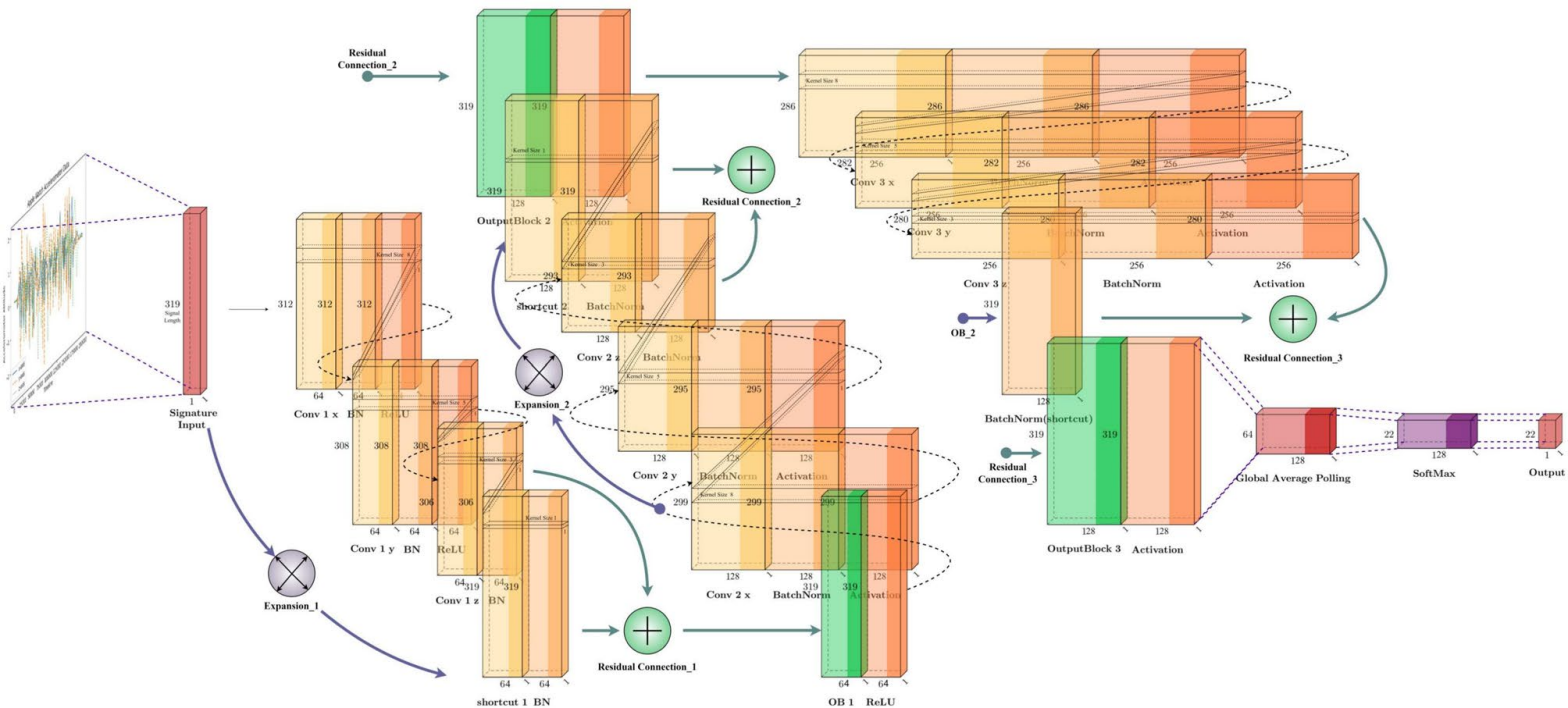
Time-series ResNet model structure.

The subsequent model explored in the experiment is ResNet^7^, which shares similarities with other traditional convolutional neural networks but distinguishes itself through the incorporation of residual blocks. The ResNet was also originally used for image classification-related tasks^75^, but it certainly shows great performance after transferring to the field of time-series classification^3^. Like FCN^5^, ResNet employs ReLU^68^ function and batch normalization^74^, and replaces the final fully connected layer with a global average pooling layer. Similarly, ResNet can utilize class activation maps^56^. Illustrated in Fig. 13, the introduction of residual connections between blocks addresses the issue of gradient vanishing commonly encountered in deep convolutional networks^76^. ResNet comprises three residual blocks, each containing three convolutional layers interconnected via residual connections. while the number of filters doubles from 64 in the initial block, the kernel size remains consistent at 8, 5, and 3 for each convolutional layer, and concludes with SoftMax^65^ for classification

### InceptionTime

**Fig. 14:**
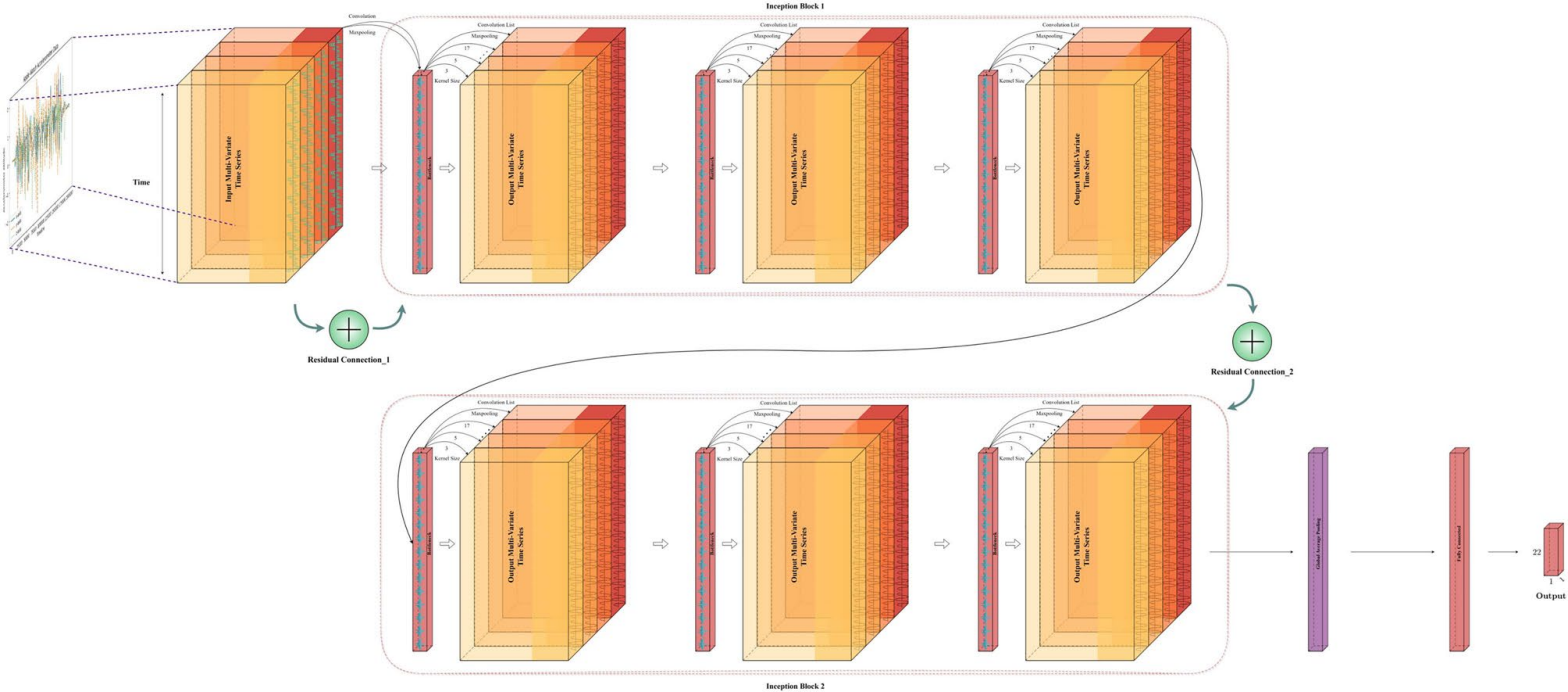
Time-series inception-time model structure.

The final model explored in the experiment is InceptionTime^12^, a new architecture tailored for time-series classification, particularly effective for small datasets^3^ like in-air signatures as shown in section 6 model selection part. The inception module was originally proposed in GoogLeNet (inception-v1) for image recognition that uses kernels of various sizes to extract dedicated features^77^. There are various derivatives of the inception^74^, and the main idea of Inception modules is to use the kernel of various sizes to capture features of different scales and apply the $1\times 1$ kernel to reduce the number of parameters, then use global average pooling to reduce overfitting^78^. As illustrated in Fig. 14, InceptionTime comprises two primary blocks, each containing three inception modules and linked by two residual connections. One residual connection links the input to the first block, and the other connects the first block to the second block, indicated by green arrows in the diagram. These residual connections help mitigate the gradient vanishing problem just like ResNet^7^. Each of the two blocks includes three inception modules, containing three bottleneck processes and three inception layers. The architecture incorporates batch normalization^74^ and ReLU^68^ for activation. In contrast to ResNet^4,7^ and FCN^4,5^, InceptionTime features both a fully connected layer and global average pooling layers. The final classification is performed using SoftMax^65^. Within each inception module, there are 3 main components listed as follows:

- Bottleneck layer: The first component is the bottleneck layer, a key innovation that reduces the dimensionality of the input multivariate signature signals to a smaller channel size by $1\times 1$ kernel. This dimensionality reduction is crucial for managing the computational complexity and preventing the model from overfitting to the training data. In the bottleneck process, the stride and kernel sizes are both set to 1, ensuring that the input data spatial dimensions are preserved while reducing the number of channels.
- Sliding filters: The second component involves sliding filters. In this stage, filters with kernel sizes of [3, 5, 8, 11, 17] process the output from the bottleneck layer independently and in parallel. These sliding filters capture different features of the time-series data at various temporal resolutions, which is essential for accurately classifying complex patterns in the data.
- Max-pooling operation: The third component involves a max-pooling operation that, different from traditional methods applied after convolutional layers, functions independently on the input signal. This independent operation ensures that the pooling process does not interfere with the feature extraction performed by the sliding filters. The results from the max-pooling are then concatenated with the sliding convolution kernel outputs. The next inception module will receive the information and go through the process again.

Each of the two blocks in the architecture contains three inception modules, resulting in three bottleneck processes and three layers of inception modules. This deep, layered approach allows the model to capture intricate patterns in the time-series data by progressively refining its feature extraction process.

### Justification for model selection

The selection of seven deep learning models-Fully Convolutional Network (FCN), Multilayer Perceptron (MLP), ResNet, Encoder, Multi-Channel Deep Convolutional Neural Network (MCDCNN), Time-CNN, and InceptionTime-is based on their effectiveness in time-series classification and their suitability for in-air signature recognition. Below, we outline how each model addresses the key challenges of this task.

#### Handling high dimensionality

In-air signature data captures multiple sensor dimensions, incluiding accelerometer, gyroscope, and attitude sensors, over time, requiring models capable of processing multivariate time-series data.

- MCDCNN: Processes each sensor dimension separately before integrating features, preserving individual characteristics while modeling interactions.
- InceptionTime: Uses multi-scale convolutional filters to capture features across different temporal resolutions.
- FCN: Extracts spatial and temporal features directly through convolutional layers, reducing the need for feature engineering.

#### Capturing temporal dependencies

Temporal dynamics are critical in in-air signatures, where the sequence and timing of gestures must be preserved.

- ResNet: residual connections enable deep networks to capture long-range dependencies effectively.
- Encoder: self-attention mechanisms capture contextual relationships between distant time points.
- Time-CNN: lightweight 1D convolutional layers efficiently extract sequential features.

#### Robustness across devices

Variability in sensor placement, sensitivity, and user interaction necessitates models that generalize well across different devices.

- FCN: translation-invariant convolutional layers handle variations in sensor data.
- MCDCNN: separately processes sensor dimensions, allowing adaptation to device-specific differences.
- InceptionTime: multi-scale feature extraction accommodates variations in gesture speed and duration.

#### Computational efficiency

Real-world applications require a balance between accuracy and efficiency for scalability and low-latency processing.

- Time-CNN: a lightweight architecture optimized for real-time inference.
- MLP: serves as a computationally efficient baseline, evaluating the complexity of the data.

#### Model diversity for comprehensive evaluation

The selected models represent a diverse range of architectures, ensuring a thorough exploration of effective approaches.

- FCN: spatial and temporal feature extraction.
- MLP: nonlinear feature learning.
- ResNet: deep hierarchical learning.
- Encoder: long-range dependencies.
- MCDCNN: multivariate processing.
- Time-CNN: efficient temporal modeling.
- InceptionTime: multi-scale feature learning.

#### Proven performance in time-series classification

All models have demonstrated state-of-the-art performance on benchmark time-series datasets, making them strong candidates for in-air signature recognition.

- FCN, ResNet: achieved top results in UCR/UEA time-series classification benchmarks.
- InceptionTime: outperformed traditional methods and deep learning models^12^.
- MCDCNN: designed specifically for multivariate time-series classification.

By leveraging these models, we ensure a comprehensive evaluation of in-air signature recognition, identifying the most effective architecture for this task.

## Embodied cognition inspired feature selection

The final goal of cognition computation is to design a holistic algorithmic structure that could completely imitate the human cognition process^33^. Deep learning models based on CNN certainly show the possibility of imitating and understanding human cognition in an algorithmic way^79^. In our research, we mainly aim to use model selection and dimension-wise Shapley Value feature selection to mimic the human cognition process by analyzing the contribution of each device and each sensor dimension during the in-air signature recognition process in a systematic way.

Signature signing is a complicated process with the involvement of multiple muscle groups and conceptual understandings^80^. Sophisticated human physical movement will not be possible without enough cognition abilities^81^. Therefore, by delving into the contribution of each dimension during the in-air signature recognition process, we could better understand and mimic the weighting of each muscular group and how much our cognition values them respectively in a complicated human cognition task.

**Fig. 15:**
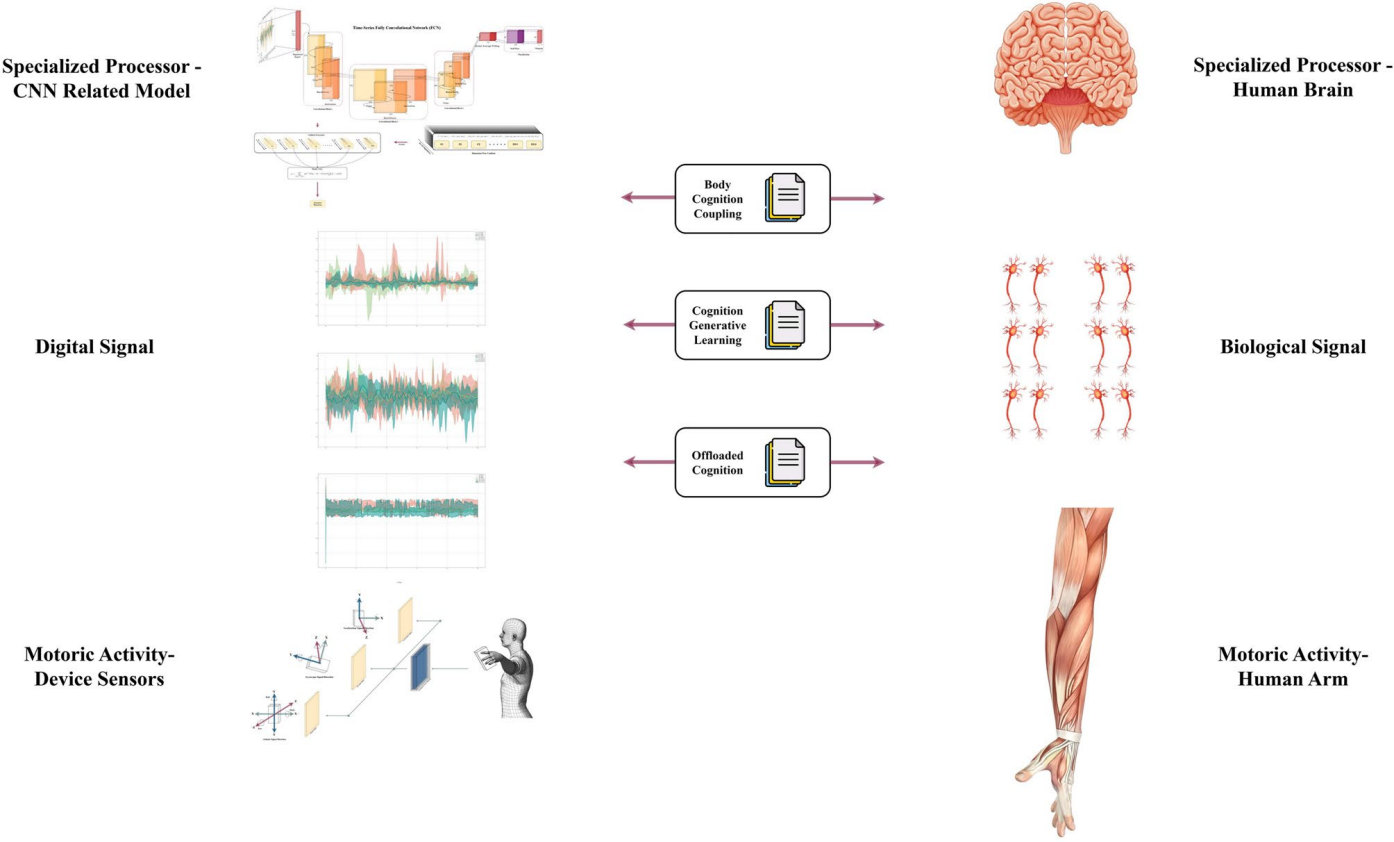
The similarity between the human movement cognition process and our in-air signature dimension-wise feature selection approach.

As shown in the Fig. 15, generally speaking, there are two stages that get involved with human cognition during the physical movement, the converting of motoric movement into biological neuron signals, and the sending back of biological information to brain^82^. In our experiment, the sensors embedded in the smartwatch and mobile phone convert the movement information into digital signals and finally transmit to CNN-related models with dimension-wise Shapley Value feature selection analysis.

The supporting evidence for the similarity of the human signature signing process using muscle movement and the sensing from digital signal sensors could be found in Diaz Moises et al.’s research^32^. They designed an anthropomorphic robotic arm to mimic the human signature signing process and used reverse kinematics to calculate the angular parameters from the robotic arm as extended features to fuse them into other online signature datasets, which has achieved cutting-edge performance for online signature verification^32^. Additionally, recent research strongly indicates that creature brain connectomes have the nature of nested recurrent loops, which are highly similar to modern convolutional neural networks^83^.

In the past decades, various cognition theories have been proposed aiming to build a theoretical foundation to better explain the human cognition process, including the embodied cognition^84^, ecological cognition^85^, the distributed cognition^86^, and etc. We finally decided to apply embodied cognition to our experiment as it has been used in various research to explain the relationship between human cognition and physical movement^87^, especially in the hand gesture and handwriting research field^88^, and its theoretical application has been used to discuss the issues get involved with the newly emerging Virtual-Reality (VR) techniques^89^. Therefore, in this section, in order to show that our approach mimics the human movement cognition process, we will first discuss our proposed in-air signature dimension-wise Shapley Value feature selection in detail and build correlation among our experiment pipeline and three popular embodied cognition theoretical perspectives^84^, including the Cognition and Body Movement Coupling^88^, Cognition Generative Learning^90^, and the Offloaded Cognition^91^.

### Dimension-wise Shapley value

In modern machine learning models, the Shapley Value finds diverse applications and extensions in feature selection and data evaluation^92^. Initially introduced by Lloyd S. Shapley^93^, this fundamental Game Theory concept addresses profit distribution among cooperating participants. The Shapley Value is underpinned by four axioms-Dummy Player, Symmetry, Additivity, and Efficiency-that ensure fair distribution within a coalition^93^. These axioms, represented in Eqs. 1, 2, 3, and 4, also form the basis for our use of Shapley Values in dimension-wise coalition analysis. Given a set of players $P = \{1, 2, \ldots , i, \ldots , n\}$ and a coalition $S\subseteq \lbrace 1 \ldots p\rbrace \{i\}$, with $v$ being the characteristic function that maps $v(S)$ to $\Re$, the following axioms apply.

**Theorem 1** *Axiom of dummy player: a player who does not add any value to the coalition’s profit will not receive any share of the profit.*

1 $$\begin{aligned} \begin{array}{l} \text {If } v(S \cup \{i\})=v(S) \forall S, \\ \text {Then } \varphi _{P_i}(v)=0 \end{array} \end{aligned}$$

**Theorem 2** *Axiom of symmetry: if two players contribute equally to the profit of any coalition, they should receive the same Shapley Value distribution.*

2 $$\begin{aligned} \begin{array}{l} \text {For } P_i,P_j \in P, \\ \text {If } v(S \cup \{P_i\})=v(S \cup \{P_j\}) \forall S, \\ \text {Then }\varphi _i(v)=\varphi _j(v) \end{array} \end{aligned}$$

**Theorem 3** *Axiom of additivity: if a player engages in several separate games independently, and these games are merged into a larger one, the Shapley Value assigned to this player in the larger game should be the aggregate of their Shapley Values across each individual game.*

3 $$\begin{aligned} \begin{array}{l} \text {For } P_i \in P, \\ v(S)\rightarrow \Re , w(S)\rightarrow \Re , \\ \varphi _{P_i}(v+w)=\varphi _{P_i}(v)+\varphi _{P_i}(w) \end{array} \end{aligned}$$

**Theorem 4** *Axiom of efficiency: each player’s Shapley Value should correspond to a profit combination that equals the total profit generated by the entire game.*

4 $$\begin{aligned} & \sum _{P_i \in P} \phi _{P_i}(v)=v(P) \end{aligned}$$

5 $$\begin{aligned} & \varphi _{i} =\displaystyle \sum \limits _{S\subseteq \lbrace 1 \ldots p\rbrace \{i\}} (p!)^{-1}|S|!|p-|S|-1|![val(S\bigcup \{i\})-val(S)] \end{aligned}$$

**Fig. 16:**
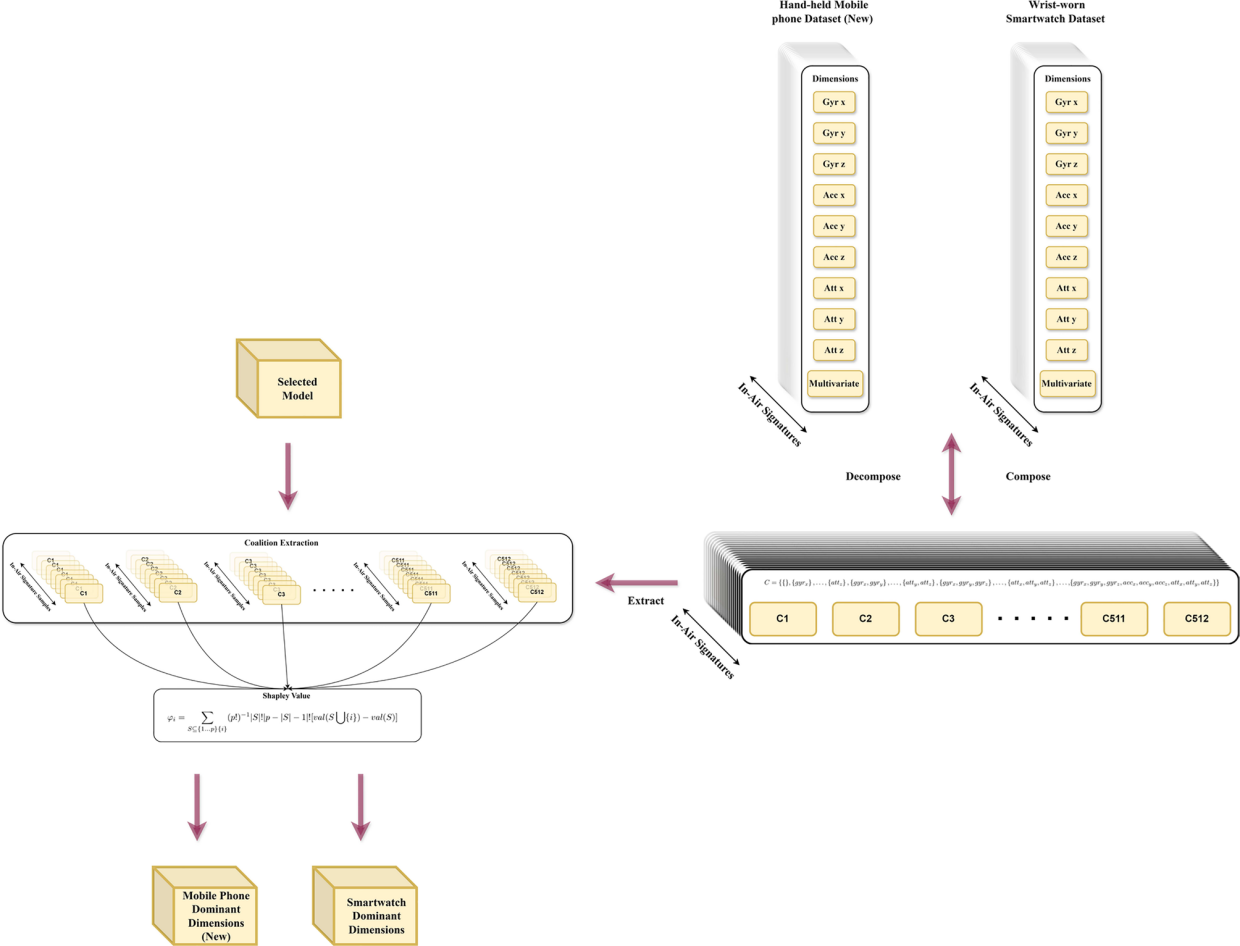
Dimension-wise Shapley value feature selection procedure demonstration.

The formula 5^93^ illustrates the computation of $\varphi _{i}$, the Shapley Value for player *i*. Here, $S\subseteq \lbrace 1 \ldots p\rbrace \{i\}$ denotes each coalition that player *i* could potentially join. The term (*p*!) denotes the total number of possible coalitions among *p* players, while |*S*|! indicates the number of ways to form a specific coalition, and $|p-|S|-1|!$ signifies the number of ways other players can join after player *i*. Thus, $(p!)^{-1}|S|!|p-|S|-1|!$ represents the weight for a particular coalition. Additionally, $val(S\bigcup \{i\})-val(S)$ calculates the marginal contribution of player *i* in coalition *S*. Summing up the weighted marginal contributions across all potential coalitions yields the Shapley Value for player *i*. As shown in Fig. 16, in our context, 9 dimensions of the online in-air signature dataset signals gathered by 3 embedded sensors are akin to 9 player *i* participating in the game of classifying online in-air signatures into the correct subject. We will conduct this dimension-wise Shapley Value feature selection across 2 devices, wrist-worn smartwatches, and hand-held mobile phones (MIAS-427), and perform comparative analysis in “Experiment”.

#### Example calculation

**Fig. 17:**
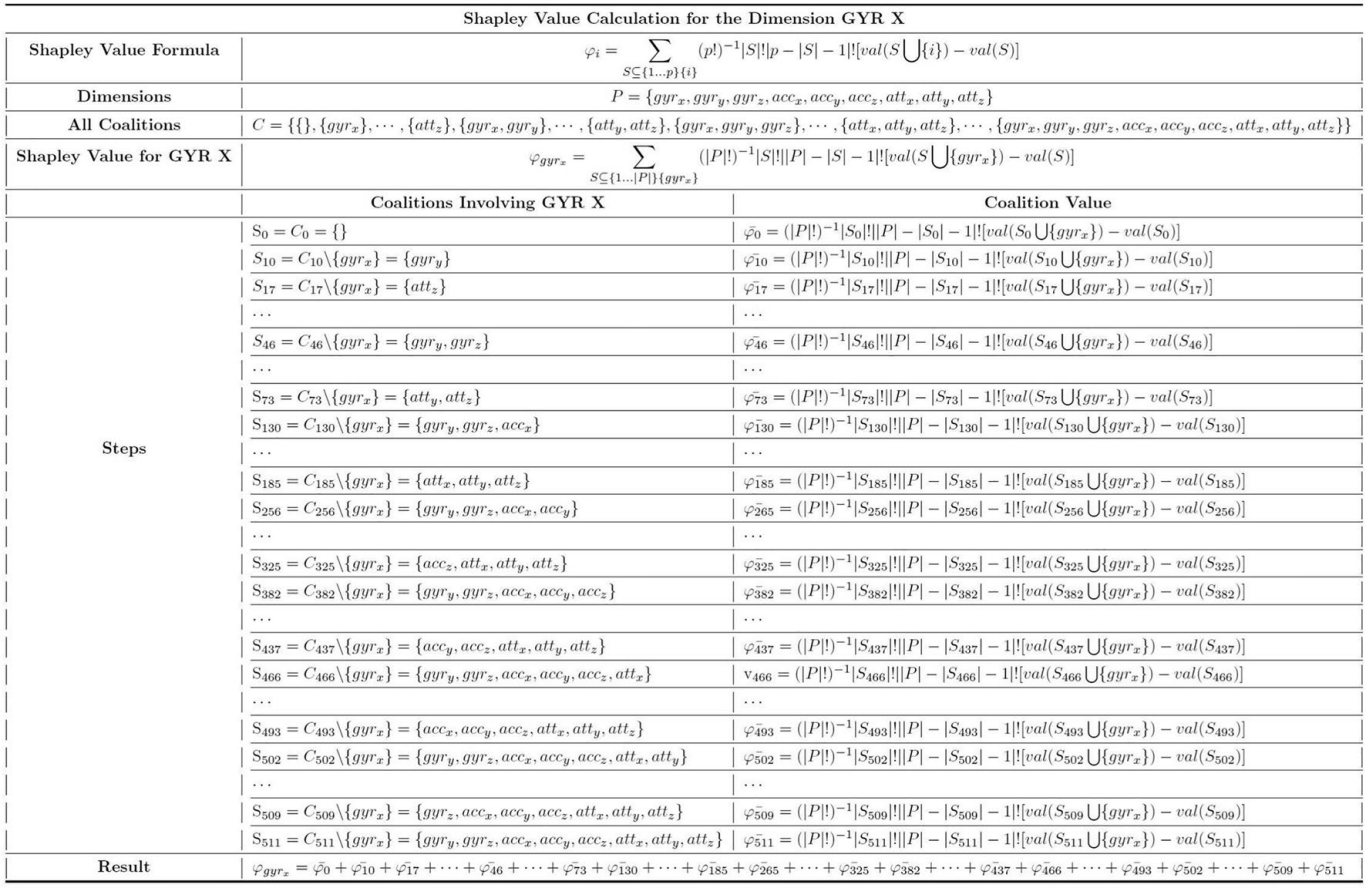
The dimension-wise Shapley value calculation for online in-air signature with steps.

To better demonstrate the calculation process, let’s consider an example for the dimension $gyr_x$, the x dimension is collected by the gyroscope, as depicted in Fig. 17. Under the *Dimensions* header, all the dimensions involved in the classification are listed, while *C* represents all possible coalition of the 9 dimensions, including the none set. In the computation of $\varphi _{gyr_x}$, we iterate through every coalition in which $gyr_x$ could potentially participate. For each coalition, $gyr_x$ is excluded to form coalition *S*, and then the coalition value is computed. Finally, these values are summed up to yield the Shapley Value for $gyr_x$ as $\varphi _{gyr_x}$.

### Embodied cognition

Embodied cognition, as one of the most influential human cognition theories, suggests that cognition is not only limited to the human brain but is also formed by body experiences and interactions with environmental devices^84^. In this subsection, inspired by the embodied cognition principles, we explore how cognition extends beyond the brain to include interactions with the body and surroundings during the in-air signature recognition process. By applying generative learning concepts^90^, we demonstrate how our approach using model selection and dimension-wise Shapley Value feature selection simulates human cognitive processes by following a similar signature recalling mechanism. Additionally, we explore the concept of offloaded cognition^94^ and discuss how offloaded cognition, as a cognition buffer, plays a similar role in both human cognition and our replicated process. Drawing from the embodied cognition theory, we illustrate how human cognition disperses cognitive processing across motoric movements just as our approach distributes recognition accuracy among sensor dimensions, providing valuable insights into this intricate relationship.

#### Cognition and body movement coupling

Human cognition happens within the interaction among the brain, body, and surroundings^88^, and the interaction with the external object could enhance the human cognition process^95^. Our dimension-wise Shapley Value feature selection was inspired by this principle by incorporating two environmental devices that interact closely with human gestures. We apply feature selection to 9 dimensions of in-air signature from both the hand-held mobile phones and wrist-worn smartwatches^1^ with 3 embedded motion sensors respectively and isolate the dimension-wise feature contributions to replicate the interaction of human gesture with the external devices and environmental factors.

#### Cognition generative learning

Generative learning in the field of human cognition refers to the process of establishing the connection between human cognition and past experiences^90^. A similar process could be found in our experiment as the aforementioned deep learning models in “Time-series deep learning model structures”, ResNet^4^, with the residual connection structure. The experiment result of ResNet can be found later in “Experiment”, in which we dedicate a section to show the model selection process with 7 popular time-series CNN models. Generative learning stemming from physical movements is an important part of embodied cognition^88^. Hand gestures and the use of interactive devices for handwritten characters are crucial components in terms of the formation of human cognition^88^. While generative learning could be frequently found in human handwritten processes^96^. In our experiment, during the dataset collection phase, data are generated from hand gesture movement by participants recalling their past handwritten habits. This data-generative process from embedded sensors and CNN model recognition replicates the human cognition generative learning process.

#### Offloaded cognition

Using physical movement as the cognitive buffer during the human cognition process is called offloaded cognition^94^. Offloaded cognition plays an important role in human cognition as it makes the cognition process more efficient, reliable, and consistent^97^. Offloaded cognition theory suggests that using external devices or objects to perform gestures could transfer part of the human cognition process into the targeted devices^88^. In our experiment, just as gesturing can partially offload cognition from the brain onto the hands^95^, dimension-wise Shapley Value feature selection replicates this procedure and distributes cognitive processing across 3 different dimensions of 3 different sensors from 2 external devices respectively based on their importance in the replicated cognition process.

#### Theoretical grounding of Shapley value in embodied cognition

Embodied cognition theory posits that cognition is not confined to the brain but is distributed across the body and its interactions with the environment^84^. This perspective provides a compelling framework for understanding how sensor dimensions contribute to in-air signature recognition, as it mirrors the way humans rely on physical movements and environmental interactions to perform cognitive tasks.

#### Implications for in-air signature recognition

By applying model selection and dimension-wise Shapley Value feature selection, our experiment pipeline mimics the principles of embodied cognition. Just as human movements and interactions with the environment influence cognitive processes, our experiment considers how different dimensions of sensor data contribute to the recognition of online in-air signatures, which is a complicated human cognition process, thereby replicating the ideology from the perspective of embodied cognition that cognition is not solely confined to the brain but is also distributed across the body and environment.

## Experiment

**Fig. 18:**
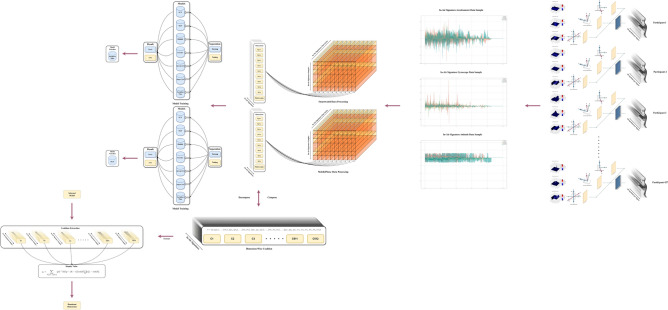
Experiment pipeline. The experiment has data collection phase, data analysis phase, model selection phase, and dimension-wise Shapley value feature selection phase.

Our experiment consists of three primary steps as shown in Fig. 18. The process moves from right to left, starting with data collection, followed by data processing and model selection. It includes a dimension-wise comparison using Shapley Values and also incorporates instance pattern visualization using class activation mapping (CAM)^56^.

- Data processing: this phase involves data interpolation and truncation, the division into training and testing sets, and the combination of dimension-wise coalitions.
- Model selection: during this stage, we assess the recognition of in-air signature signals using both univariate and multivariate approaches. First, each dimension of the in-air signature is analyzed as an independent uni-variate time series. Then, the nine dimensions are integrated into a multivariate time series. This methodology helps us empirically identify the most effective model for recognizing in-air signatures on both smartwatches and mobile devices.
- Dimension-wise Shapley value feature selection: this section focuses on identifying key dimensions that significantly impact in-air signature recognition. We evaluate whether such dominant or non-dominant dimensions exist, identify them, and quantify their contribution to recognition accuracy compared to other dimensions across smartwatch and mobile phone devices.

### Data processing

**Fig. 19:**
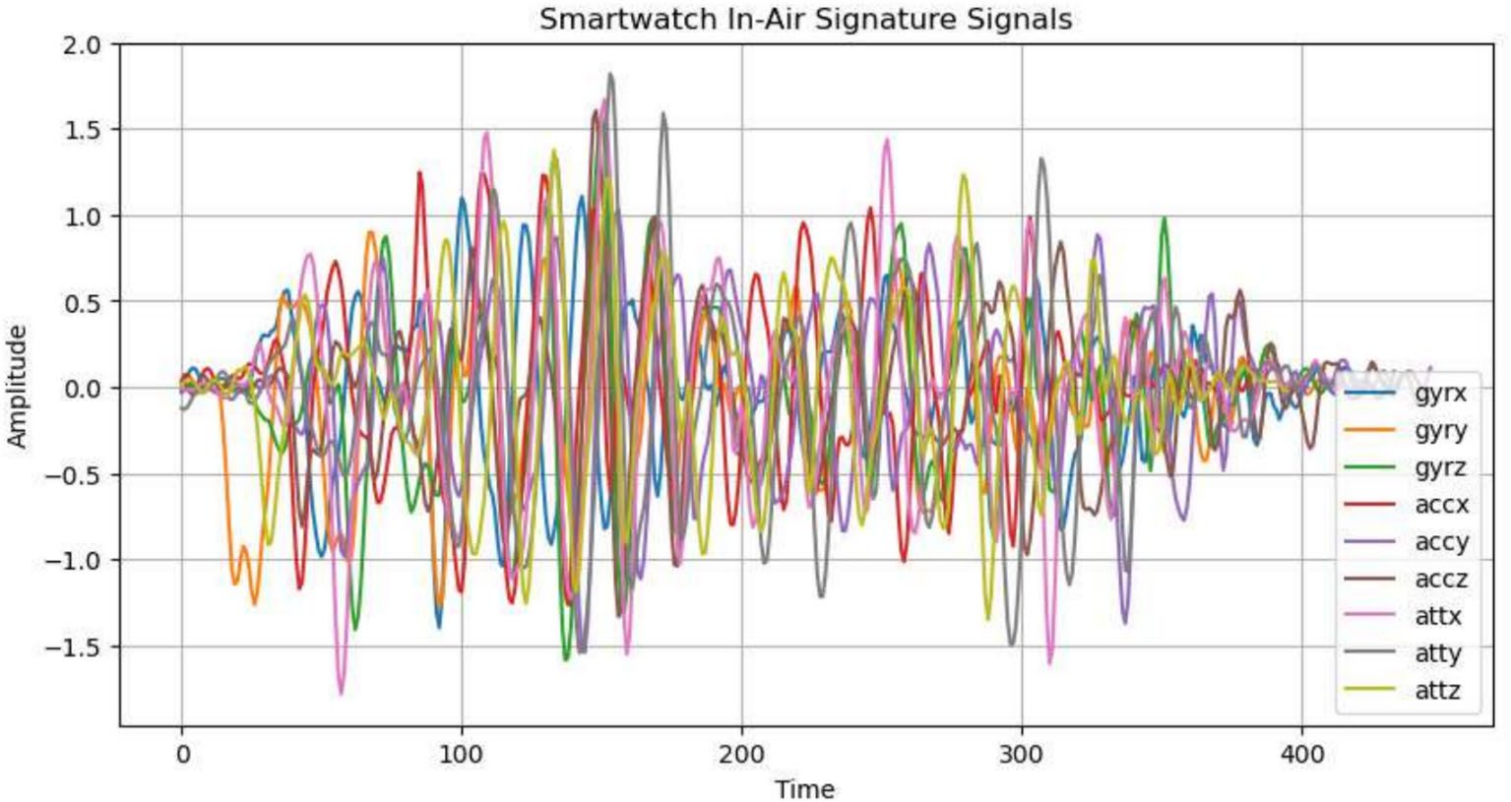
The 9-dimensional in-air signature time-series for the same signature.

Compared to the numerous hand-written signature datasets available^54^, the in-air signature research community lacks a substantial dataset for use. Therefore, there are two in-air signature datasets used. For the mobile phone in-air signatures, we use the newly collected MIAS-427 dataset. For the smartwatch, we use the in-air signature dataset created by Li, who collected in-air signatures from 22 participants, with each providing 10 genuine signatures^1^. As shown in Fig. 18, for the mobile phone, we have 4270 in-air signatures, which belong to 427 subjects; while there are in total 220 smartwatch in-air signatures which belong to 22 subjects, and each signature has nine dimension time series data information. The headers of the Tables 5 and  4 shows the nine dimensions of the smartwatch and mobile phone sensors, which includes data from three sensors and three orientations for each: gyroscope (x, y, z), accelerometer (x, y, z), and attitude (x for pitch, y for roll, and z for yaw). The consistency of the two datasets makes device-wise comparison for in-air signatures possible. As we can observe in the Fig. 19, this figure demonstrates the 9 dimensions for one signature, each dimension has its own characteristics and patterns.

After that, for both the univariate and multivariate analysis, the data is divided into the training and testing set, with 20% being testing and 80% being training. Each uni-variate and multi-variate in-air signature time series dataset for smartwatch and mobile phone are fed into 7 models, as discussed in “Time-series deep learning model structures”, separately for the analysis, which means we have 140 experiments here (i.e. (9 uni-variate + 1 multivariate) $\times$ 7 models $\times$ 2 devices). For the dimension-wise coalition, we divide each 9-dimensional in-air signature signal into 512 dimension combinations (i.e. $2^9$) based on C in Fig. 16, which gives us 2,998,880 signatures signals in total (i.e. 512 $\times$ 220 + 512 $\times$ 4270), and then we extract the signature with the same dimension combination as one experiment, which means we have 1024 experiments here (i.e. $512 \times 2$ experiment for coalitions across 2 device dataset), and for each experiment (coalition), we have 20% of the signature set for testing and 80% for training. In this way, we can divide each dimension into independent and mutually dependent channel sets (coalition) for dimension-wise Shapley value^93^ feature selection as shown in Fig. 16, which is also considered as a systematic ablation experiment, and we aim to find out which dimension contributes the most to the in-air signature recognition performance across devices.

**Table 4:** Mobile phone in-air signature sample data.

Mobile phone in-air signature sample for subject 1 signature 1								
$Acc_x$	$Acc_y$	$Acc_z$	$Gyr_x$	$Gyr_y$	$Gyr_z$	$Ori_x$	$Ori_y$	$Ori_z$
$-0.44313$	$-8.78151$	4.868727	$-10.784$	0.039824	$-5.61297$	2.446671	$-1.38939$	$-18.3264$
$-0.48447$	$-8.56189$	3.485494	$-12.9766$	$-0.40324$	$-2.60337$	0.356771	1.152709	$-2.50698$
$-0.61656$	$-8.61197$	2.981159	$-13.828$	$-0.51613$	$-1.65867$	$-0.28343$	1.931627	2.336218
$-0.77201$	$-8.78855$	3.000329	$-13.7056$	$-0.46182$	$-1.93873$	$-0.28343$	1.931627	2.336218
$-0.88342$	$-8.94843$	3.187611	$-12.9766$	$-0.40324$	$-2.60337$	$-0.28343$	1.931627	2.336218
$-0.88342$	$-8.94843$	3.187611	$-11.9682$	$-0.46815$	$-2.97202$	$-0.28343$	1.931627	2.336218
$-0.72352$	$-8.68269$	2.726951	$-10.8453$	$-0.6436$	$-3.00261$	$-0.28343$	1.931627	2.336218
$-0.43086$	$-8.19468$	1.860314	$-9.73243$	$-0.88144$	$-2.81264$	$-0.28343$	1.931627	2.704359
$-0.05149$	$-7.56522$	0.724398	$-8.75415$	$-1.13354$	$-2.51963$	$-0.28343$	1.931627	2.985879
...	...	...	...	...	...	...	...	...

**Table 5:** Smartwatch in-air signature sample data.

Smartwatch in-air signature sample for subject 1 signature 1								
$Acc_x$	$Acc_y$	$Acc_z$	$Gyr_x$	$Gyr_y$	$Gyr_z$	$Ori_x$	$Ori_y$	$Ori_z$
0.006494	0.009239	0.01121	0.086356	0.029508	0.011415	0.45407	0.70815	0.070161
$-0.00074$	0.01422	0.001814	0.088319	0.029273	0.027723	0.455669	0.708618	0.070167
-0.00668	0.013277	-0.00159	0.066002	0.026148	0.058603	0.456869	0.709155	0.070393
$-0.00844$	0.017108	0.001519	0.037187	0.023975	0.072369	0.457283	0.70961	0.070874
-0.00716	0.013046	-0.00435	0.019389	0.026953	0.083881	0.457445	0.710026	0.071477
0.003638	0.006969	$-0.00419$	0.010959	0.020459	0.093629	0.457578	0.710534	0.072157
0.011639	0.002621	-0.00286	0.029967	0.015235	0.077567	0.457893	0.710999	0.072829
0.012988	0.001942	$-0.01286$	0.070353	0.018348	0.053111	0.45838	0.711309	0.073376
0.01261	0.007601	-0.01088	0.115285	0.029243	0.035008	0.459069	0.711681	0.073653
...	...	...	...	...	...	...	...	...

### Model selection

**Fig. 20:**
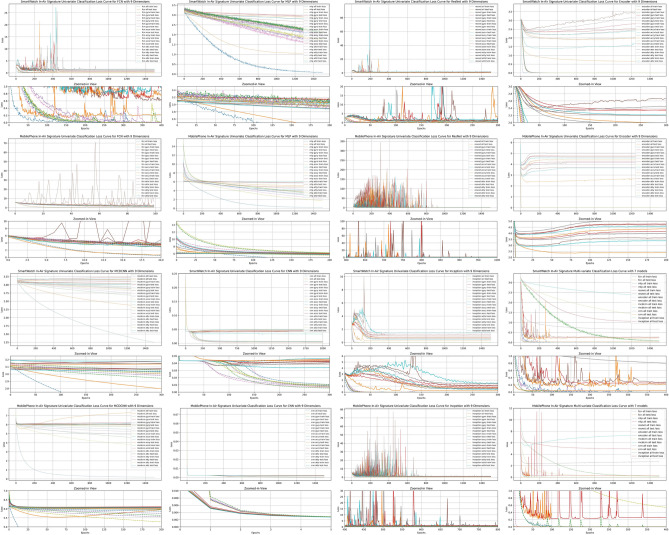
Loss curve for each model with respect to 9 dimensions of the mobile phone and smartwatch in-air signature signals.

To mitigate the influence of the model on dimension-wise Shapley Value feature selection for each dimension, we aim to empirically identify the optimal model for the smartwatch in-air signature dataset^1^ and the MIAS-427 respectively. To ensure the robustness of each model, we fine-tuned them, with hyper-parameters detailed in Table 6. Figure 20 presents the loss curves for seven models across nine dimensions. The figure is organized by different models, with each dimension indicated in the legend for each model’s loss curve. The training loss is depicted with dashed lines, while the testing loss is shown with solid lines. These curves allow us to observe the training pattern variations for each model concerning different in-air signature dimensions. Additionally, the subplot in Fig. 20 illustrates the zoomed-in in-air signature training patterns for the seven models.

**Table 6:** Hyper-parameters for model selection.

Hyper-parameters for model selection			
Model	Optimizer	Learning rate	Epoches
FCN	Adam	$1\times 10^{-5}$	1500
MLP	Adadelta	$1\times 10^{-5}$	1500
ResNet	Adam	$1\times 10^{-5}$	1500
Encoder	Adam	$1\times 10^{-5}$	1500
MC-DCNN	SGD	$1\times 10^{-5}$	1500
Time-CNN	Adam	$1\times 10^{-5}$	1500
InceptionTime	Adam	$1\times 10^{-5}$	1500

**Fig. 21:**
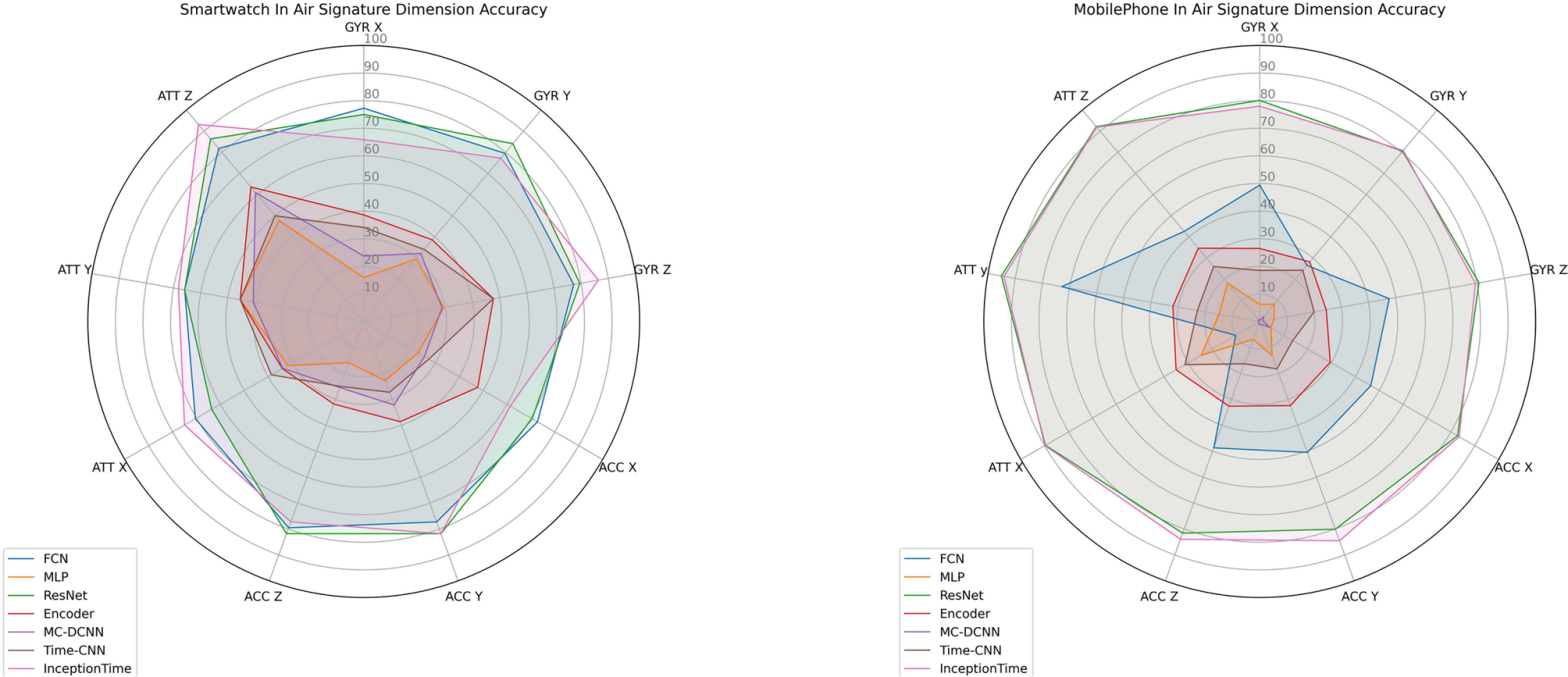
The accuracy radar chart for 9 uni-variate dimensions without Shapley Value evaluation for 7 models across two devices.

Table 7 displays numerical metrics such as training loss, validation loss, training accuracy, and validation accuracy for the seven models across nine dimensions with respect to two devices. The table highlights the highest validation accuracy in bold for each dimension and model. As we could observe, for both the two device in-air signature data, the best recognition result is generally obtained by FCN, ResNet, and InceptionTime models. Figure 21 better illustrates this phenomenon that, for the smartwatch in-air signatures, testing accuracy results converge towards the ATT Z dimension, suggesting its significant impact on accuracy. For the MIAS-427 dataset in-air signatures, the ResNet and Inception perform well consistently across all uni-variate dimension signals, while the FCN only shows better performance when multi-variate in-air signatures are taken into consideration.

However, in collaborative machine learning tasks involving nine dimensions for a multivariate time series, pinpointing each dimension’s exact contribution to overall performance poses challenges. This underscores the relevance of the Shapley Value methodology^93^, which fairly determines each dimension’s influence by considering their interactions, as detailed in the next dimension-wise Shapley Value feature selection subsection. By utilizing the dimension-wise Shapley Value analysis with the optimal model for the smartwatch and mobile phone in-air signatures respectively, we could determine the most dominant feature dimension in the in-air signature recognition process and analyze the motoric preference of human cognition while doing complicated cognitive tasks like signatures.

**Table 7:** In air signature dimension-wise result with respect to 7 models.

		FCN				MLP				ResNet				Encoder			
		Train Loss	Val Loss	Train Acc	Val Acc	Train Loss	Val Loss	Train Acc	Val Acc	Train Loss	Val Loss	Train Acc	Val Acc	Train Loss	Val Loss	Train Acc	Val Acc
$gyr_x$	Smartwatch	0.000863	1.143057	1.000000	**0.772727**	2.041270	2.901309	0.534091	0.159091	0.000027	1.049522	1.000000	0.750000	0.046418	2.463133	1.000000	0.386364
	MobilePhone	1.690352	2.055047	0.597775	0.494145	3.513643	5.743859	0.329333	0.062061	0.000088	1.011116	1.000000	**0.800937**	0.000000	4.753933	1.000000	0.264637
$gyr_y$	Smartwatch	0.000946	0.631819	1.000000	0.795455	1.867170	2.596801	0.596591	0.295455	0.000030	0.597382	1.000000	**0.840909**	0.048767	2.296226	1.000000	0.386364
	MobilePhone	1.971687	3.566267	0.530738	0.265808	3.733193	5.459705	0.305328	0.081967	0.000087	1.092465	1.000000	0.805621	0.000000	4.671137	1.000000	0.282201
$gyr_z$	Smartwatch	0.000276	0.869979	1.000000	0.772727	1.949114	2.754028	0.590909	0.295455	0.000017	0.701116	1.000000	0.795455	0.044129	2.029505	1.000000	0.477273
	MobilePhone	1.515104	2.047502	0.644028	0.476581	3.949366	5.720282	0.272248	0.052693	0.000129	0.987692	1.000000	**0.806792**	0.000000	4.214752	1.000000	0.326698
$acc_x$	Smartwatch	0.000822	1.405717	1.000000	**0.727273**	2.065331	2.772530	0.505682	0.227273	0.000026	1.777774	1.000000	0.704545	0.049346	2.282771	1.000000	0.477273
	MobilePhone	1.344280	2.159226	0.679742	0.464871	3.312011	6.112179	0.326112	0.046838	0.000121	0.996195	1.000000	0.827869	0.000000	4.570889	1.000000	0.295082
$acc_y$	Smartwatch	0.000253	0.765759	1.000000	0.772727	2.078427	2.872400	0.500000	0.227273	0.000023	0.855328	1.000000	**0.818182**	0.057834	2.352815	1.000000	0.386364
	MobilePhone	1.515113	1.947253	0.618560	0.504684	2.673995	5.124926	0.432084	0.131148	0.000124	1.127218	1.000000	0.800937	0.000000	4.394173	1.000000	0.324356
$acc_z$	Smartwatch	0.000491	0.743038	1.000000	0.795455	2.020146	2.950209	0.551136	0.159091	0.000018	0.741366	1.000000	**0.818182**	0.051929	2.455319	1.000000	0.318182
	MobilePhone	1.338269	2.048572	0.678571	0.487119	3.124808	5.884278	0.362998	0.069087	0.000134	0.967641	1.000000	**0.816159**	0.000000	4.214752	1.000000	0.326698
$att_x$	Smartwatch	0.000794	1.341715	1.000000	0.704545	1.588894	2.338112	0.647727	0.318182	0.000088	1.341873	1.000000	0.636364	0.061780	2.396163	1.000000	0.340909
	MobilePhone	0.920287	12.018706	0.789520	0.100703	2.966992	3.702783	0.359192	0.244731	0.000044	0.686136	1.000000	0.896955	0.000000	3.700962	1.000000	0.350117
$att_y$	Smartwatch	0.001008	1.610170	1.000000	0.659091	1.385714	2.207671	0.704545	0.454545	0.000067	1.610684	1.000000	0.659091	0.048654	2.372221	1.000000	0.454545
	MobilePhone	0.657939	1.138778	0.866218	0.728337	2.184671	4.566315	0.526054	0.149883	0.000039	0.469704	1.000000	**0.950820**	0.000000	4.117480	1.000000	0.319672
$att_z$	Smartwatch	0.001030	0.905462	1.000000	0.818182	1.632002	2.057175	0.619318	0.477273	0.000027	0.623890	1.000000	0.863636	0.045538	1.536219	1.000000	0.636364
	MobilePhone	1.227948	2.319797	0.711944	0.426230	1.725388	4.106923	0.599532	0.181499	0.000066	0.481198	1.000000	**0.921546**	0.000000	3.655498	1.000000	0.346604
Multi-variate	Smartwatch	0.000164	0.218313	1.000000	0.954545	0.056910	1.037466	1.000000	0.750000	0.000228	0.268284	1.000000	**0.977273**	0.000000	0.663066	1.000000	0.886364
	MobilePhone	0.014021	0.206765	0.997951	**0.980093**	0.154127	4.934531	0.970726	0.254098	0.000012	0.225528	1.000000	0.978923	0.000000	2.535954	1.000000	0.523419

		MC-DCNN				Time-CNN				InceptionTime							
		Train Loss	Val Loss	Train Acc	Val Acc	Train Loss	Val Loss	Train Acc	Val Acc	Train Loss	Val Loss	Train Acc	Val Acc				
$gyr_x$	Smartwatch	0.000116	5.097457	1.000000	0.237288	0.003358	0.044626	0.926136	0.340909	0.000111	1.178935	1.000000	0.659091				
	MobilePhone	5.751400	6.042672	0.028409	0.005319	0.000463	0.002160	0.808841	0.185012	0.000195	0.964016	1.000000	0.779859				
$gyr_y$	Smartwatch	0.000106	4.814487	1.000000	0.322034	0.005424	0.046393	0.880682	0.340909	0.000101	0.564286	1.000000	0.772727				
	MobilePhone	4.823625	6.036449	0.173514	0.022163	0.000433	0.002076	0.822600	0.242389	0.000120	1.108800	1.000000	**0.809133**				
$gyr_z$	Smartwatch	0.000087	5.190854	1.000000	0.288136	0.003616	0.041996	0.920455	0.477273	0.000108	0.528887	1.000000	**0.863636**				
	MobilePhone	4.426040	6.291317	0.185315	0.010638	0.000519	0.002219	0.785422	0.162763	0.000113	0.957068	1.000000	**0.839578**				
$acc_x$	Smartwatch	0.000103	5.026496	1.000000	0.254237	0.006715	0.049867	0.852273	0.272727	0.000133	1.462102	1.000000	0.613636				
	MobilePhone	3.587413	6.254947	0.310752	0.042553	0.000532	0.002284	0.780738	0.138173	0.000099	0.987797	1.000000	**0.834895**				
$acc_y$	Smartwatch	0.000107	5.008159	1.000000	0.322034	0.006457	0.048751	0.857955	0.272727	0.000117	0.731059	1.000000	**0.818182**				
	MobilePhone	5.426049	5.986765	0.062063	0.011525	0.000447	0.002171	0.820258	0.182670	0.000137	0.952626	1.000000	**0.845433**				
$acc_z$	Smartwatch	0.000087	5.732913	1.000000	0.254237	0.005683	0.044177	0.875000	0.250000	0.000130	0.780274	1.000000	0.772727				
	MobilePhone	4.426040	6.291317	0.185315	0.010638	0.000519	0.002219	0.785422	0.162763	0.000113	0.957068	1.000000	**0.839578**				
$att_x$	Smartwatch	0.000270	7.360557	1.000000	0.338983	0.004650	0.047987	0.897727	0.386364	0.000148	0.981134	1.000000	**0.750000**				
	MobilePhone	6.028891	6.051734	0.003059	0.004433	0.000502	0.001988	0.819672	0.312646	0.000082	0.676804	1.000000	**0.899297**				
$att_y$	Smartwatch	0.000090	5.778140	1.000000	0.406780	0.003616	0.041204	0.920455	0.454545	0.000140	1.456859	1.000000	**0.681818**				
	MobilePhone	5.856832	6.056956	0.020979	0.005319	0.000425	0.002096	0.828454	0.229508	0.000076	0.510279	1.000000	**0.941452**				
$att_z$	Smartwatch	0.000178	3.349710	1.000000	0.610169	0.002841	0.033254	0.937500	0.500000	0.000129	0.438309	1.000000	**0.931818**				
	MobilePhone	5.858071	6.032607	0.020542	0.007979	0.000341	0.002071	0.864754	0.259953	0.000134	0.547519	1.000000	0.919204				
Multi-variate	Smartwatch	1.245477	2.308463	0.957265	0.440678	0.041054	0.042022	0.289773	0.113636	0.000508	0.186687	1.000000	**0.977273**				
	MobilePhone	0.193026	7.452806	0.991259	0.102837	0.000220	0.001851	0.915105	0.341920	0.000020	0.290013	1.000000	0.973068				

#### Comparison of model accuracy across devices

To analyze the impact of device type on model performance, we compare accuracy across different feature sets for the smartwatch and mobile phone. Table 8 summarizes the best-performing models for each feature dimension and device type.

Overall, the results indicate that the mobile phone consistently achieves higher accuracy for most feature dimensions. Specifically, for accelerometer-based features (*accx*, *accy*, and *accz*), InceptionTime on the mobile phone outperforms all other models, achieving accuracies of 0.8349, 0.8454, and 0.8396, respectively. In contrast, the smartwatch yields slightly lower performance, with ResNet achieving the highest accuracy for *accy* and *accz* at 0.8182.

For gyroscope-based features (*gyrx*, *gyry*, and *gyrz*), the smartwatch and mobile phone exhibit varying strengths. Notably, InceptionTime on the smartwatch achieves the best performance for *gyrz* (0.8636), while ResNet on the mobile phone performs best for *gyrx* and *gyrz* at 0.8009 and 0.8068, respectively. The smartwatch demonstrates competitive accuracy for *gyry* (0.8409 with ResNet), but the mobile phone outperforms it with InceptionTime at 0.8091.

Regarding angular velocity features (*attx*, *atty*, and *attz*), the smartwatch exhibits a distinct advantage in *attz*, where InceptionTime achieves 0.9318, surpassing the mobile phone’s best model (ResNet, 0.9215). However, for *attx* and *atty*, the mobile phone significantly outperforms the smartwatch, with ResNet achieving 0.9508 for *atty* compared to the smartwatch’s 0.6818.

In the multi-variate setting, combining all features yields the highest performance across both devices. The best-performing models are ResNet for the smartwatch (0.9773) and FCN for the mobile phone (0.9801), highlighting the effectiveness of deep learning models when leveraging comprehensive motion data.

These findings suggest that while mobile phones generally provide superior accuracy, smartwatches can still achieve competitive performance, particularly in gyroscopic and angular velocity-based features.

**Table 8:** Summary of best models across devices.

Dimension	Smartwatch (best model)	MobilePhone (best model)
*gyrx*	FCN (0.7727)	ResNet (0.8009)
*gyry*	ResNet (0.8409)	InceptionTime (0.8091)
*gyrz*	InceptionTime (0.8636)	ResNet (0.8068)
*accx*	FCN (0.7273)	InceptionTime (0.8349)
*accy*	ResNet (0.8182)	InceptionTime (0.8454)
*accz*	ResNet (0.8182)	InceptionTime (0.8396)
*attx*	InceptionTime (0.7500)	InceptionTime (0.8993)
*atty*	InceptionTime (0.6818)	ResNet (0.9508)
*attz*	InceptionTime (0.9318)	ResNet (0.9215)
Multi-variate	ResNet (0.9773)	FCN (0.9801)

Additionally, the final row of Table 7 showcases metrics specific to multivariate in-air signatures. While, even though, the FCN does not perform well compared with other models like ResNet and InceptionTime for uni-variate signals, it outperforms any other models when multiple channels are taken into consideration with 98% accuracy. Remarkably, the ResNet model^4^ and InceptionTime^12^ achieve the highest validation accuracy for smartwatch multivariate in-air signature time series, both reaching 97.73%. With FCN being the best model for mobile phone in-air signatures (MIAS-427); Ultimately, we choose InceptionTime for smartwatch and for conducting dimension-wise Shapley Value analysis of in-air signatures for three primary reasons:

- InceptionTime consistently provides the best (and most stable) validation scores for most smartwatch uni-variate in-air signature dimensions.
- InceptionTime achieves the highest validation scores for multivariate in-air signatures, on par with ResNet.
- InceptionTime is a newly proposed architecture specifically designed for time series with small training sizes^12^.

Consequently, in the subsequent dimension-wise Shapley Value Feature Selection section, we employ FCN for mobile phone in-air signature (MIAS-427) and InceptionTime for smartwatch in-air signature to identify the most influential dimension through mutual interactions across devices.

### Dimension-wise Shapley value feature selection

We systematically explore all possible combinations of 9 dimensions, totaling 512 coalitions (i.e., $2^9$), with the best performance model for MIAS-427 and smartwatch in-air signatures, and compute the Shapley Value for each dimension to assess their impact on performance. This approach allows us to conduct 1024 (i.e., $512 \times 2$ devices) ablation experiments, effectively identifying the most influential dimension affecting the accuracy of in-air signature recognition. As outlined earlier in the dimension-wise Shapley value feature selection “Embodied cognition inspired feature selection”, the Shapley Value method is thoroughly described. The subsequent Result analysis “Results” will present and interpret the accuracy contributions of each feature.

### Class activation map

**Fig. 22:**
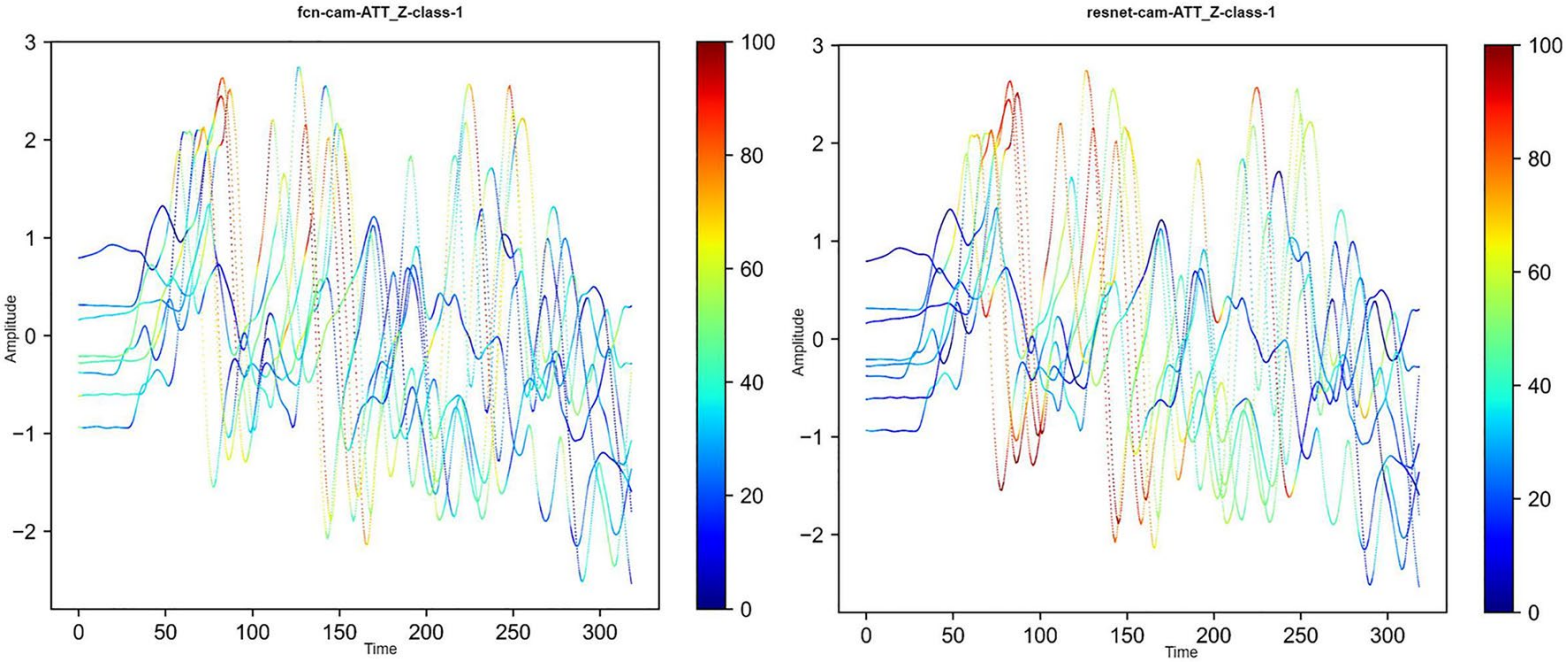
The class activation maps for the same signature with the FCN being left while the ResNet being right.

We include the Class Activation Map (CAM)^56^ to visually highlight distinctive sections within in-air signature signals. Figure 22 displays the CAM for the FCN model using the $att_z$ dimension, where the red areas are the most distinguishable and the blue areas are the least distinguishable. Additionally, the figure shows the CAM for the ResNet model with the $att_z$ dimension, revealing that these models classify the same subject based on different distinguishing characteristics. While the beginning and ending parts of the signals sometimes do provide useful information for recognizing individual in-air signatures.

## Results

**Fig. 23:**
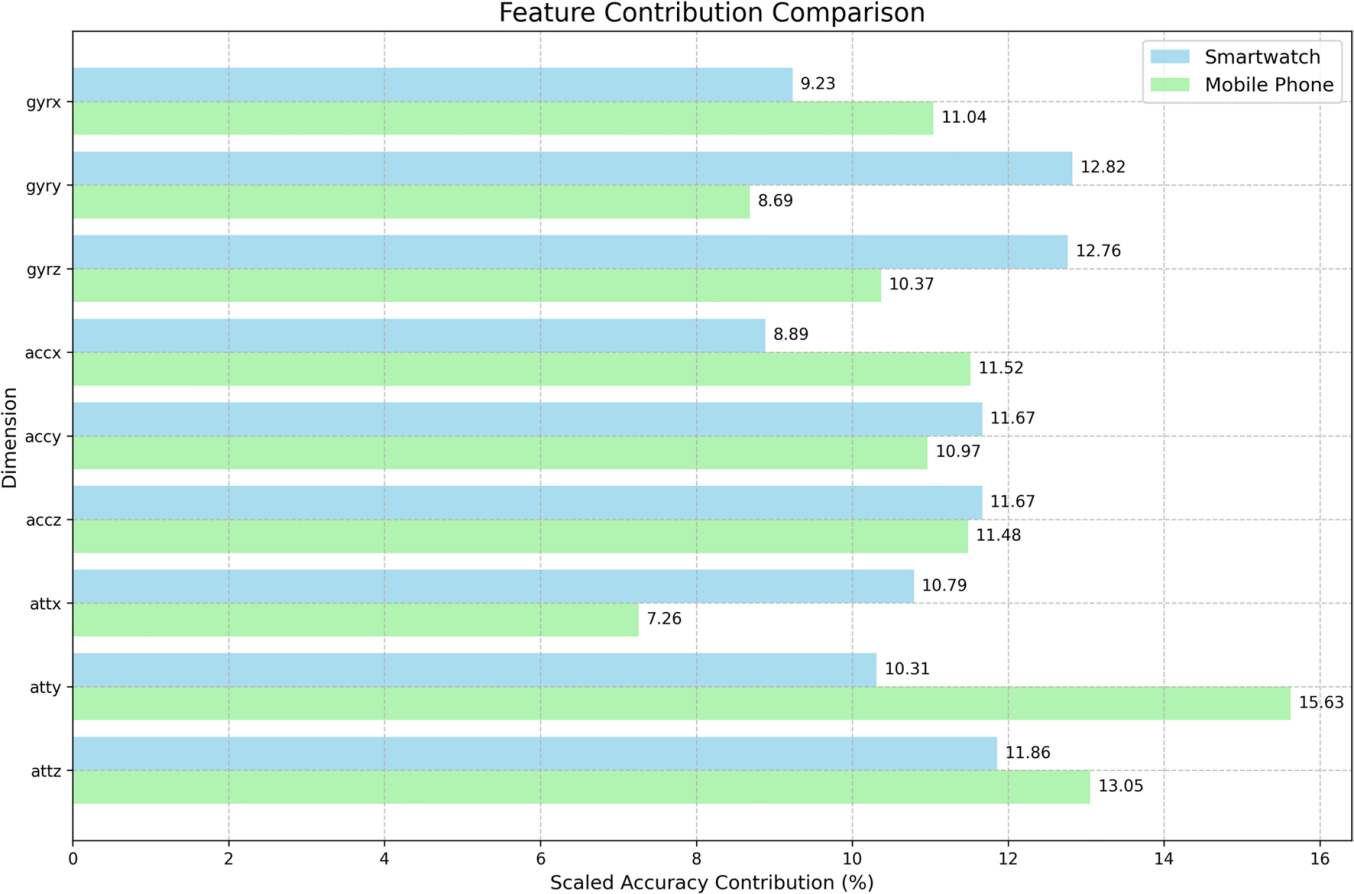
Mobile phone and smartwatch in-air signature Shapley value feature contribution.

As depicted in Fig. 23, the Shapley Values for accuracy are presented with precision to four decimal places, normalized with respect to 100% for both the FCN for mobile phone in-air signatures (MIAS-427) and InceptionTime model for smartwatch in-air signatures using two 9-dimensional multivariate in-air signature dataset. Interestingly, the $att_z$ dimension does not exhibit a dominant contribution compared to other dimensions for smartwatch in-air signatures. While each dimension is essential and contributes uniquely to accuracy, their individual impacts vary. For instance, the $gyr_z$ and $gyr_y$ dimensions demonstrate stronger contributions, accounting for 12.76% and 12.82% respectively (normalized to 100%), in terms of accuracy, which is 4% higher compared to the $acc_x$ dimension. This disparity highlights that $acc_x$ provides the lowest Shapley Value contribution. Similarly, as we can observe the $att_y$ dimension contributes the most to the mobile phone in-air signature dataset with 15.63% accuracy which is 8.37% higher compared with $att_x$. This result reveals the human motoric preferences when doing the cognition-related task with different external devices, and provides statistical support and parameter tuning evidence when creating a human cognition computation machine that mimics complicated human cognitive behavior.

These findings provide crucial insights into human motoric behavior when performing cognition-related tasks with different external devices. The varying importance of feature dimensions highlights the need for device-specific dimension-wise Shapley Value feature selection and parameter tuning in in-air signature recognition systems. Specifically:

- For smartwatch-based models, gyroscope features should be prioritized in model design and preprocessing pipelines due to their dominant contribution.
- For mobile phone-based models, accelerometer features-particularly atty-should be emphasized in feature engineering to enhance accuracy.
- For cross-device models, hybrid approaches leveraging a combination of both dominant feature sets may yield optimal results.

**Fig. 24:**
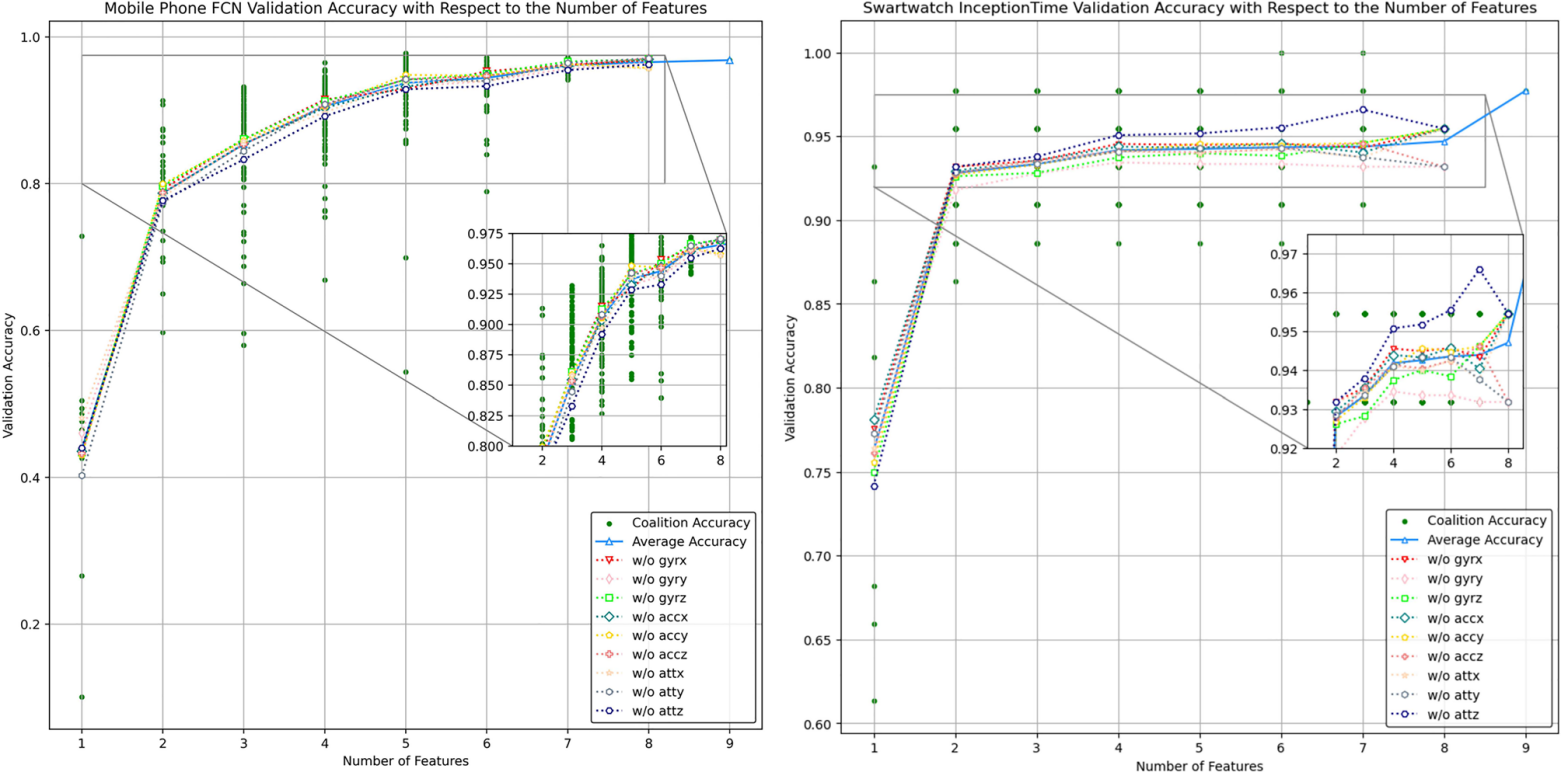
Validation accuracy with respect to the number of features for mobile phone (MIAS-427) and smartwatch in-air signatures.

Our research also evaluates accuracy across different numbers of features, as depicted in Fig. 24. The green dots represent coalition accuracy for each feature combination, showcasing distinct values amidst overlapping points. Meanwhile, the solid blue curve illustrates the mean accuracy as the number of features increases, indicating improved accuracy with more information integrated into the model.

Moreover, dashed lines in the figure indicate dimension compatibility-how each dimension contributes when included or excluded in feature groups. In the zoomed-in section of Fig. 24, it’s evident that the $gyr_y$ and $gyr_z$ dimensions exhibit superior compatibility, as accuracy decreases noticeably when these dimensions are omitted for smartwatch in-air signatures; while $att_y$ and $att_z$ dimension shows better compatibility for mobile phone in-air signatures (MIAS-427). Those observations align with their higher Shapley Values of 12.82% ($gyr_y$) and 12.76% ($gyr_z$) respectively for smartwatch; while, 15.63% ($att_y$) and 13.05% ($att_z$) for mobile phone. Interestingly, we could observe the almost exact opposite dimension-wise feature compatibility for smartwatch and mobile phone in-air signature datasets as shown in Fig. 24, which indicates that human motoric preferences may differ a lot when interacting with different external devices when doing complicated cognitive tasks.

In this study, seven deep learning models were tested on a smartwatch in-air signature dataset, including FCN, MLP, ResNet, Encoder, MC-DCNN, Time-CNN, and InceptionTime. The InceptionTime model achieved the highest validation score of 97.73%, matching ResNet, for smartwatch multivariate in-air signatures, and performed consistently well on uni-variate dimensional signatures. While for the mobile phone in-air signatures (MIAS-427), the FCN model achieves the best recognition accuracy of 98%. Through dimension-wise Shapley Value feature selection using Shapley Values, it was determined that for smartwatch in-air signatures $gyr_y$ and $gyr_z$ dimensions play crucial roles, whereas $acc_x$ showed the lowest Shapley Value contribution at 8.71%. For mobile phone in-air signature (MIAS-427), the $att_y$ dimension contributes the most for the in-air signature recognition tasks of 15.63% while the $att_x$ dimension contributes the least of 7.26% accuracy.

## Research limitations and future work

Even though our newly collected mobile phone in-air signature dataset MIAS-427 is considered, currently, one of the largest datasets in the field of in-air signature recognition and even in the field of time-series classification, we are planning to shift our research direction to the in-air signature verification field and add the corresponding forgery signatures to the dataset as supplementary data in the future. Meanwile, we are planning to solve the following questions:

- The large difference in the relative average DTW distance between two in-air signature sources indicates the in-air signatures might be closely related to individuals’ personal experiences, which needs further investigation.
- Meanwhile, given more computational resource available in the future, we aim to re-evaluate the experiment using k-fold cross-validation to validate our work in a different perspective.
- We plan to propose new method integrating transformer-based models (e.g., ViT^98^, Swin Transformer^99^) to further enhance recognition accuracy. These models’ ability to capture long-range dependencies and complex patterns aligns well with the challenges of time-series data analysis.
- The proposed methodology for in-air signature dimension-wise Shapley Value Feature Selection could be potentially applied to offline signature recognition by segmenting the handwriting signatures into different strokes and analyze the human motoric preferences when generating forgery handwriting signatures. Additionally, beyond handwriting recognition, our methodology can be adapted to other biometric authentication tasks, such as gesture recognition or keystroke dynamics, where understanding the contribution of individual features is critical for improving system performance.

## Conclusion

In our research, we collected currently one of the largest inertial in-air signature recognition datasets (MIAS-427) with 4270 multivariate in-air signature signals. We performed statistical analysis with multiple domain descriptors and DTW algorithms. We performed a thorough deep learning model selection process with 7 models in comparison with the smartwatch and MIAS-427 in-air signature dataset. Then we discussed how our approach was inspired by the embodied cognition theory with dimension-wise Shapley Value analysis. As a result, the FCN and InceptionTime give the best recognition accuracy with 98% and 97.73% for MIAS-427 and the smartwatch in-air signature dataset. For the smartwatch in-air signatures, $gyr_y$ and $acc_x$ give the best and the worst contribution with 12.82% and 8.71%; while for the MIAS-427, $att_y$ and $att_x$ give the best and the worst accuracy of 15.63% and 7.26% respectively. Meanwhile, the dimensionality compatibility also shows a reverse pattern for different external devices. Our research provides a valuable in-air signature dataset for the community while revealing the human motoric preference when getting involved with complicated cognition tasks, which enriches the current parametric evidence for building a future human cognitive computation machine with detailed motoric preferences.

## Data Availability

The datasets used and/or analysed during the current study available from the corresponding author on reasonable request.
